# Semi-solid Extrusion 3D Printing of Chitosan/Carbon Nanotube Nanocomposite Films for Microextraction of Pesticides in Water

**DOI:** 10.1021/acsomega.5c11817

**Published:** 2026-04-13

**Authors:** Giuseppe da Silva Salvador, Vítor Augusto Bauer, Rita de Cássia dos Reis Schmidt, Nadine Lysyk Funk, Sofia Aquino Monteiro, Camila Scheid, Silvio Buchner, Cesar Liberato Petzhold, Ketherin Adam Antoni, Tiago Espinosa de Oliveira, Josias Merib, Monique Deon

**Affiliations:** a Programa de Pós-Graduação em Biociências, 117303 Universidade Federal de Ciências da Saúde de Porto Alegre, Rua Sarmento Leite 245, CEP, 90050-170 Porto Alegre, RS, Brazil; b Curso de Graduação em Química Medicinal, 117303 Universidade Federal de Ciências da Saúde de Porto Alegre, Rua Sarmento Leite 245, CEP, 90050-170 Porto Alegre, RS, Brazil; c Programa de Pós-Graduação em Ciências Farmacêuticas, 28124 Universidade Federal do Rio Grande do Sul, Av. Ipiranga 2752, CEP, 90610-000 Porto Alegre, RS, Brazil; d Instituto de Física, 28124 Universidade Federal do Rio Grande do Sul, CP 15051 CEP, 91501-970 Porto Alegre, RS, Brazil; e Instituto de Química, 28124 Universidade Federal do Rio Grande do Sul, CP 15003 CEP, 91501-970 Porto Alegre, RS, Brazil

## Abstract

In this work, a novel thin-film microextraction (TF-SPME) device was developed using semi-solid extrusion 3D printing, aiming to provide a sustainable and customizable tool for the environmental monitoring of pesticides in water. The device was fabricated from a nanocomposite of chitosan (CS) and multi-walled carbon nanotubes (MWCNT) and the printing approach enabled direct shaping of the extraction films under mild conditions. Rheological evaluation confirmed that the CS-based hydrogels, with and without MWCNT, possess pseudoplastic and thixotropic behavior, suitable characteristics for extrusion-based printing. Films measuring 20 mm × 5 mm × 1 mm were designed in a geometry compatible with TF-SPME strips, and after printing, they were evaluated for the hydrolytic stability of chitosan. Both CS and CS/MWCNT films exhibited similar swelling index in water (287 ± 7 % and 244 ± 7 %, respectively) and maintained structural integrity without disintegration in either water or acetonitrile throughout the evaluation period, regardless of the presence of MWCNT. The printed devices were successfully applied to the thin-film solid-phase microextraction of organochlorine and organophosphorus pesticides in water followed by analysis using gas chromatography coupled to mass spectrometry (GC-MS). Better extraction efficiency and linear response were observed for the MWCNT-containing 3D-printed films for seven evaluated pesticides (alpha-BHC, aldrin, endosulfan I (alpha), dieldrin, 4,4-DDE, 4,4-DDD, and phorate). Molecular dynamics simulations suggest that the presence of CNT is associated with microstructural and hydration effects within the chitosan matrix, which favor interactions between the analytes and the sorbent phase. The limits of quantification were in the range 50–200 μg L^‑1^. This study highlights the potential of semi-solid extrusion 3D printing in the development of green, functional, and versatile analytical platforms for monitoring of organic pollutants in environmental samples.

## Graphical abstract

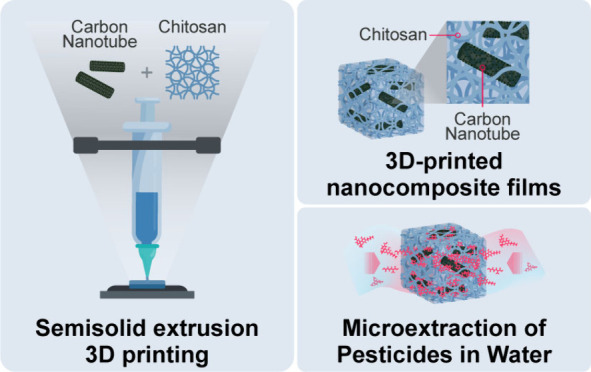

## 1 Introduction

The increasing complexity of environmental samples and the need for trace analysis have driven the development of more sensitive, selective, and sustainable analytical strategies. Sample preparation plays a crucial role in this context, particularly when analyzing emerging contaminants and pesticides, notably organochlorine and organophosphorus compounds, and consequently sample preparation techniques are increasingly being explored and developed to meet these analytical demands.^1^ Widely used in agriculture, these contaminants are frequently detected in aquatic environments and pose potential risks to human health and ecosystems. Among the available techniques for aqueous sample preparation, thin-film solid-phase microextraction (TF-SPME), a variation of classical SPME, has demonstrated feasibility for the determination of pesticides and organic pollutants.^2^

In the development of customizable analytical devices for sample preparation, 3D printing has emerged as a promising technology, offering advantages such as design flexibility, low cost, and reproducibility.^3,4^ Techniques such as stereolithography (SLA) and fused deposition modeling (FDM) have been increasingly explored for fabricating solid-phase extraction platforms tailored to specific analytical needs. For instance, Kołodziej, Sobczak, and Goryński (2022)^5^ used FDM to fabricate a polyamide-based extraction device similar to those used in TF-SPME, which required no coating as the extraction phase. Particularly, the extraction device composed of polyamide reinforced with carbon fiber was capable of directly extracting the analytes through a straightforward experimental workflow. The resulting device proved to be a low-cost, environmentally friendly, and highly efficient extraction alternative, achieving quantification limits as low as 1 μg L^–1^ when associated with LC-MS analysis for small molecules with diverse log *P* values and molecular weights.^5^ Additionally, a variation of SLA using a Liquid Crystal Display (LCD) light source was employed to develop polymeric ionic liquid-based adsorbents for TF-SPME, with quantification limits in the μg L^–1^ range for organic contaminants.^6^ More recently, a FDM 3D-printed device based on open-source electronic platform has also been proposed for the microextraction of environmental pollutants.^7^

Despite this progress, semi-solid extrusion-based 3D printing (SSE) remains largely underexplored in the context of analytical sample preparation. In this technique, a semi-solid formulation is extruded through a syringe nozzle, with the piston movement controlled by the printer. Unlike FDM and SLA, this technique operates under mild processing conditions, eliminating the need for high temperatures or photopolymerization, which allows the use of thermosensitive, biodegradable, and non-thermoplastic materials.^4^ This is particularly relevant for analytical applications given that FDM, the most popular printing technique, relies mainly on commodity thermoplastics such as PLA, ABS, PET, PP, and PE, which are generally chemically inert and lack functionalities,^8^ offering limited affinity for analyte interaction when employed as active extraction phases. The SSE printable formulations, typically hydrogel- or paste-based, can be precisely modified, enabling the direct incorporation of functional additives, such as nanomaterials, plasticizers, or pH modifiers, within a single extrusion step.^9–11^ This technique also aligns with green chemistry principles, supporting the use of renewable biopolymers^12^ while offering low-cost setup, ease of implementation, and compatibility with multicomponent formulations, an attractive alternative for the development of sustainable and high-performance devices.^4,13^ Hydrogels are compatible with SSE^14^ and have already been applied as efficient sorbent phases in various extraction formats.^9,15^ Their integration into 3D-printed devices enables the development of customized platforms for analytical sample preparation.

Among materials compatible for this approach, chitosan (CS) stands out as a natural, biodegradable biopolymer with inherent chemical functionality due to its amino and hydroxyl groups. CS has been investigated as a base material for the development of extraction phases.^16^ For 3D-printing, it can form stable hydrogels with suitable rheological properties for extrusion-based methods.^17^ Furthermore, the incorporation of nanomaterials, particularly multi-walled carbon nanotubes (MWCNT), into CS matrixes enhances the physicochemical and adsorptive properties. These nanocomposites can increase surface area, modulate hydrophobicity/hydrophilicity, and introduce new interaction mechanisms with target analytes.^18^ The development of CS-based nanocomposites modified with carbon nanomaterials has already shown promise in extraction and microextraction applications. For example, Silvestro et al. (2021) demonstrated the use of CS-graphene membrane for the extraction of organic micropollutants.^19^ Wei et al. (2023) prepared CS/CNT hydrogels for adsorption of acid red 73 in aqueous and soil environments.^20^ More recently, Lessa et al. (2025) developed CS/CNT films for adsorption of dyes from aqueous samples.^21^

Based on this perspective, the present study proposes the development of an innovative 3D-printed film microextraction device, composed of chitosan/MWCNT nanocomposite, fabricated via semi-solid extrusion 3D printing. To the best of our knowledge, there are no previous reports of 3D-printed SPME devices for microextraction of pesticides obtained by SSE technique. The proposed strategy enables the direct fabrication of functional extraction devices using hydrogel-based inks, avoiding multistep synthesis routes or the immobilization of sorbent materials onto external supports. This novel platform is applied to thin-film solid-phase microextraction (TF-SPME) of pesticides in water, followed by gas chromatography coupled with mass spectrometry (GC-MS) analysis. By combining renewable biopolymer-based nanocomposites, mild processing conditions, and geometric customization enabled by 3D printing, this approach aims to offer an alternative, green, and efficient strategy for environmental monitoring, highlighting the synergy between sustainable materials and advanced fabrication techniques in modern analytical chemistry.

## 2 Experimental Section

### 2.1 Reagents and Materials

Low molecular weight chitosan (CS) was obtained from Sigma-Aldrich (50–190 kDa, degree of deacetylation >75 %), and multiwalled carbon nanotubes (MWCNT, Baytube C 150 P) were obtained from Bayer Material Science (Leverkusen, Germany). Glacial acetic acid and sodium hydroxide were obtained from Neon (São Paulo, Brazil). Analytical-grade isopropyl alcohol (Exôdo Científica, Sumaré, Brazil), acetonitrile (Supelco, Bellefonte, USA), and ultrapure water (Milli-Q purification system, Millipore, Bedford, USA) were used as solvents. Organochlorine pesticide reference standard at a concentration of 2000 mg L^–1^ containing alpha-BHC, gamma-BHC, beta-BHC, heptachlor, delta-BHC, aldrin, heptachlor epoxide isomer B, endosulfan I (alpha), 4,4-DDE, dieldrin, endrin, 4,4-DDD, endosulfan II (beta), 4,4-DDT, endrin aldehyde, endosulfan sulfate, and methoxychlor in toluene:hexane (50:50 v-v) was purchased from Sigma-Aldrich (Wyoming, USA). Reference standard of organophosphate pesticides at a concentration of 2000 mg L^–1^ containing a mix of O,O,O-triethylphosphorothioate, thionazin, sulfotep, phorate, dimethoate, disulfoton, methyl parathion, parathion, and famphur in dichloromethane was obtained from Supelco (Bellefonte, USA). An internal Standard (IS) of 2-fluorbiphenyl at a concentration of 1000 mg L^–1^ was also acquired from Sigma-Aldrich (Wyoming, USA).

### 2.2 Hydrogel Ink Preparation

To prepare the CS hydrogel containing MWCNT, 7.0 mg of MWCNT was partially dispersed in 9.3 mL of a 2.15 % (v/v) aqueous acetic acid solution using an ultrasonic bath at 5 min intervals. Then, 140 mg of CS were added, in small portions of ∼30 mg, under ultrasonic bath, until the MWCNT were completely dispersed. Afterward, 560 mg of CS was added, totalizing 700 mg of CS at the mixture, and the mixture was homogenized using a mortar and pestle to form the gel. The gel consistency was adjusted by adding 0.5 mL of 0.1 mol L^–1^ sodium hydroxide solution, and then, after homogenization and resting for 15 min, the hydrogel, named HG-CS/MWCNT, was transferred to a plastic syringe and stored under refrigeration for at least 12 h. The mass percentage of HG-CS/MWCNT is 6.7% of CS and 0.1% of MWCNT. A hydrogel containing only CS (without MWCNT) was equally prepared, omitting only the dispersion step required for the MWCNT. This hydrogel was named HG-CS.

### 2.3 HG-CS and HG-CS/MWCNT Hydrogel Characterization

The morphology of the MWCNT was studied with transmission electron microscopy (TEM). The sample preparation procedure was dispersing 1 mg of MWCNT in 1.5 mL of isopropyl alcohol using an ultrasonic bath, and then 1 to 2 drops were added to a carbon-coated copper grid. The grid containing the sample was analyzed by using a JEM-1400 Flash microscope (JEOL, Tokyo, Japan) operating at an acceleration voltage of 120 kV.

The pH measurements were performed on samples prepared by adding 1.0 g of the hydrogel to 10 mL of water and stirring for 45 min. The measurements were performed in triplicate at room temperature using a FiveEasy F20 pHmeter (Mettler Toledo, Greifensee, Switzerland).

The hydrogels also had their rheological properties studied by an Ares G2 rheometer (TA Instruments, New Castle, DE, USA) with a 25 mm diameter parallel plate and a 1 mm gap at 25 °C. In rotational mode, a flow ramp test was conducted at shear rates ranging from 0.4 to 1.0 s^–1^. The viscoelastic properties of the hydrogels were further evaluated in oscillation mode. An oscillatory amplitude test (angular frequency of 1 Hz and stress sweep from 0.1 to 500%) was performed to determine the linear viscoelastic region (LVR) and yield point (Pa). Subsequently, an oscillation frequency test was carried out within the LVR (strain of 5% and angular frequency range of 0.1 to 600 rad s^–1^) to assess the storage modulus (*G*′), loss modulus (*G*″), and tangent delta (tan δ). Finally, a three-step oscillation method was applied to evaluate the thixotropy by alternating strains of 2.5% (within the LVR) and 250% (beyond the yield point) over five cycles of 60 s each. All analyses were performed in triplicate.

### 2.4 3D Printing of Films from Hydrogel Inks

The films were printed using the hydrogel inks previously prepared using a BioEnder semi-solid extrusion 3D-printer (BioEdTech, São Paulo, Brazil) with the following parameters: extrusion speed of 8 mm s^–1^; room extrusion temperature; 50% infill percentage; number of layers equal to 3; nozzle diameter of 0.41 mm (conical nozzle, 22G); and dimensions and shape as shown in Figure 1A, established in the PrusaSlicer software. The printed films were subjected to drying in an oven at 60 °C for 30 min and then immersed in a 1.0 mol L^–1^ sodium hydroxide solution for 24 h. Afterward, the films were washed with ultrapure water to remove sodium hydroxide excess, gently dried with soft absorbent paper, and then stored in a desiccator. The films obtained from the hydrogels HG-CS and HG-CS/MWCNT were named TF-CS and TF-CS/MWCNT, respectively.

**Figure 1:**
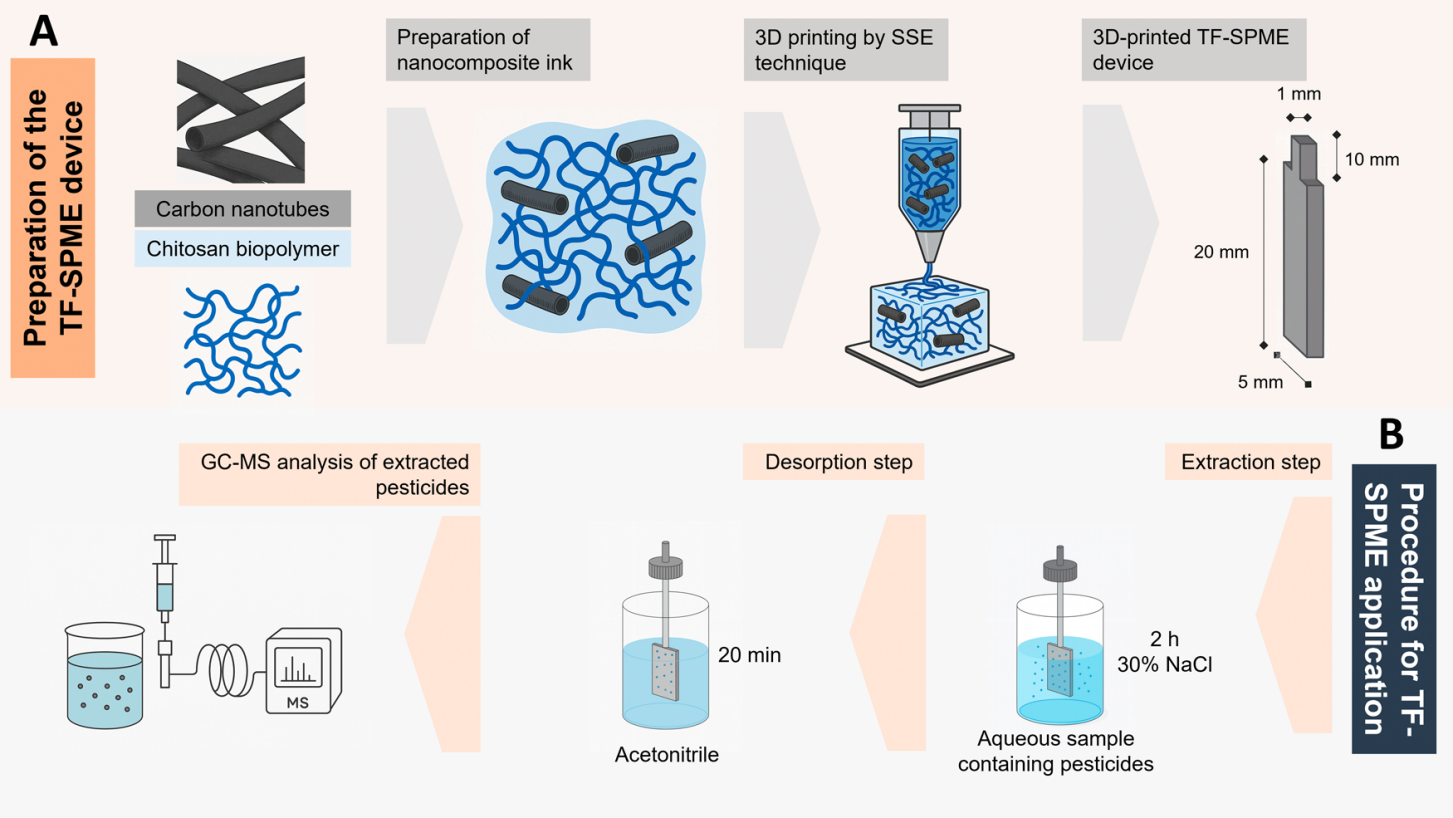
Illustration for the preparation of 3D-printed nanocomposite TF-SPME devices (A) and procedure for its use in extraction and analysis of pesticides (B).

### 2.5 3D-Printed Films TF-CS and TF-CS/MWCNT Characterization

The films mean mass (*n* = 9) was measured on an analytical balance, and the real dimensions (length, width, and height) were measured on the films immediately after printing (wet) and on the films after their complete preparation (dry), using a digital caliper.

The loss on drying analysis was performed to monitor the initial drying process of the films immediately after printing. The percentage loss on drying was calculated using eq 1, where *W*_i_ is the film initial mass immediately after printing (in g) and *W*_f_ is the film mass after drying at 60 °C (in g).

1 $$\mathrm{Loss on Drying (\%)}=\frac{\left(W_{i}-W_{f}\right)}{W_{i}}\times 100$$

Scanning electron microscopy (SEM) was used to investigate the dispersion of the MWCNT within the chitosan matrix of the 3D-printed films. Dry samples were mounted on aluminum stubs using double-sided conductive carbon tape and coated with a thin gold layer using a BAL-TEC SCD 050 Sputter Coater apparatus. The images were acquired using a Zeiss EVO MA10 microscope at a magnification of 50,000× and an accelerating voltage of 10 kV.

The films swelling and deswelling index were evaluated before and after NaOH 1.0 mol L^–1^ solution treatment. For the swelling index, the films had their initial masses recorded and then individually immersed in glass containers with 5.0 mL of ultrapure water. After 1 min, the film was removed from water; excess water was gently removed with soft paper, and the mass was measured again. This process was repeated until the film disintegrated or reached a constant mass. The swollen films were evaluated for deswelling index by repeating the same procedure previously described but using acetonitrile as solvent. The swelling percentage was calculated using eq 2, where *W*_i_ is the film initial mass and *W*_f_ the final mass measured after being immersed in the solvent.

2 $$\mathrm{Swelling (\%)}\;=\;\frac{\left(W_{f}-W_{i}\right)}{W_{i}}\times 100$$

The film’s thermal behavior was studied through thermogravimetric analysis (TGA), using a TGA-50 equipment (Shimadzu, Kyoto, Japan) with a 60 °C preheating step and heated from room temperature to 900 °C, at a rate of 20 °C min^–1^, under an air atmosphere. Chitosan raw material (CS-RM) was also studied under the same conditions.

### 2.6 Instrumental Conditions for Pesticide Analysis

The chromatographic analysis of organochlorine and organophosphorus pesticides was carried out using a gas chromatograph coupled with a mass spectrometer (GC-MS) GC-MS-QP 2010 Plus (Shimadzu, Kyoto, Japan), and with an autosampler model AOC-20i (Shimadzu, Kyoto, Japan). The analysis parameters were based on a study previously reported.^22^ The NST-5MS (NST, São Carlos, Brazil) column (30 m × 0.25 mm × 0.25 μm film thickness) was used for chromatographic separation. Ultrapure helium at 1.5 mL min^–1^ was used as the carrier gas. The injection temperature was set to 250 °C, and the initial oven temperature was set at 70 °C, increased to 180 °C at a rate of 25 °C min^–1^, and increased again to 300 °C at a rate of 5 °C min^–1^ (kept for 1 min). Automated injection of 1 μL using a split ratio of 1:2 was adopted. The mass spectrometer was used in electron ionization mode (EI) at 70 eV. The ion source was kept at 250 °C, and the interface temperature was kept at 280 °C. The analytes were evaluated using selected ion monitoring mode (SIM), with the greater intensity of *m*/*z* ratio used for quantification. Retention time, m/z ratios monitored, and chemical structure of the analytes, and IS are shown in Table S1 of the Supporting Information.

### 2.7 3D-Printed TF-CS and TF-CS/MWCNT Studies in Pesticide Extraction

The 3D-printed TF-CS and TF-CS/MWCNT were evaluated in terms of their extraction efficiency using aqueous samples spiked with the 26 analytes at a concentration of 500 μg L^–1^. For the extraction efficiency evaluation, multiple films were individually fixed on the pins of a 96-well plate coupled to an orbital stirrer and then inserted into aqueous solutions containing all the analytes and the IS (at concentration of 200 μg L^–1^), at room temperature and moderate agitation. After the extraction time (evaluated from 30 to 150 min), the films were subjected to a desorption step, by immersing them into 400 μL of acetonitrile for 20 min. The organic extracts were analyzed by GC-MS as described in 2.6.

This study comprises two main steps: first, the evaluation of the extraction efficiency and method optimization; followed by the evaluation of the analytical parameters of merit which includes the assessment of a calibration curve to examine the linearity provided by the proposed methodology. After method optimization and assessment of analytical parameters, additional experiments were performed to investigate the stability and reusability of the films, followed by the analysis of the real samples.

#### 2.7.1 Evaluation of the Extraction Efficiency and Optimization of the Experimental Conditions

The performance of TF-CS and TF-CS/MWCNT films were evaluated using the extraction conditions as follows: extraction time of 120 min, NaCl concentration of 30 % (w/v), analytes concentration of 500 μg L^–1^. An IS solution of 200 μg L^–1^ was used.

In order to optimize the experimental conditions of the experimental workflow, extraction time and NaCl concentration (w/v) were evaluated in five (30, 60, 90, 120 and 150 min) and three (0, 15 and 30 %) levels, respectively, according to a Doehlert design, with triplicate in the central point (Table S4). The geometric means of the chromatographic peak areas were used to generate the statistical model, and the data were analyzed using Statistica 10 (StatSoft) software.

#### 2.7.2 Linearity Evaluation

After the best extraction device for this method (TF-CS or TF-CS/MWCNT) was established considering extraction efficiency and optimized conditions, the 3D-printed films TF-CS/MWCNT were used to assess the linear response of the compounds examined in this study. The analytical figures of merit were determined by internal standard calibration curves of each compound consisting of 5 concentration points (within 50–700 μg L^–1^) with analyses performed in triplicate. The coefficients of determination (*r* ^2^) were calculated based on the calibration curves. The limits of quantification (LOQ) for each analyte were experimentally determined through extractions in triplicate, considering coefficients of variation of ≤20% as satisfactory. The LOQ was defined as the lowest concentration within the linear range that meets this criterion. The extraction response was normalized using the ratio of the chromatographic peak area of each analyte to film individual weight since the weight directly affects the extraction efficiency.

#### 2.7.3 Stability, Reusability and Applicability in Real Samples

Storage stability of the TF-CS/MWCNT was evaluated by comparing the extraction performance of films stored for 21 days in a desiccator with that of freshly prepared films. Both stored and fresh films were subjected to extraction experiments under identical and optimized conditions, using an aqueous solution containing the analytes at a concentration of 500 μg L^–1^. Reusability was assessed by reusing TF-CS/MWCNT films in subsequent extraction cycles without any intermediate treatment. The applicability of the method to real samples was evaluated using two lake water samples collected in the metropolitan region of Porto Alegre, identified as PA and PB. Prior to analysis, the *in natura* samples were filtered to remove coarse particulate matter. The samples were then analyzed in *in natura* conditions (PA-1, PA-2, PA-4, PB-1, PB-2, PB-4) and after being spiked with the target analytes at a concentration of 500 μg L^–1^ (PA-3 and PB-3).

#### 2.7.4 Computational Methods

Molecular dynamics (MD) simulations were performed to provide mechanistic support for the extraction behavior of the CS/MWCNT. In this study, all-atom MD simulations were employed using the GROMACS 2023.2 package.^23,24^ The CHARMM36 force field^25^ was used to describe 4,4-DDD, combined with the TIP3P (transferable intermolecular potential with three points) water model^26,27^ and the INTERFACE force field^28^ for the CNT model. The structures of 4,4-DDD and the CNT were built using the CHARMM-GUI web server^29^, employing Ligand Reader & Modeler^30^ and Nanomaterial Modeler^31^, respectively. The temperature was set to 298.15 K using the velocity-rescale thermostat^32^ with a coupling constant of 1.0 ps.

Simulations were performed at constant pressure (1.0 atm) using a Parrinello-Rahman barostat^33^ with a coupling time of 5.0 ps. Nonbonded interactions were treated with a 1.2 nm cutoff, and long-range electrostatic interactions were calculated using the Particle Mesh Ewald (PME) method.^34^ All bonds involving hydrogen atoms were constrained using the LINCS algorithm.^35^ Production simulations consisted of 200 ns runs, and only the last 25 ns were used to compute structural and dynamical descriptors and interaction energies.

To quantify the coating effect with a compact thermodynamic indicator, it was used a standard Linear Interaction Energy (LIE) estimate,^36,37^ in which the mean short-range electrostatic and van der Waals interactions of the analyte with its environment are defined as ⟨*U*_elec_⟩ = ⟨Coul­(SR)_4,4‑DDD:CS_⟩ + ⟨Coul­(SR)_4,4‑DDD:Water_⟩ + ⟨Coul­(SR)_4,4‑DDD:CNT_⟩ and ⟨*U*_vdW_⟩ = ⟨LJ­(SR)_4,4‑DDD:CS_⟩ + ⟨LJ­(SR)_4,4‑DDD:Water_⟩ + ⟨LJ­(SR)_4,4‑DDD:CNT_⟩. The relative free-energy indicator between coatings is then written as ΔΔ*G*_LIE_ = α­[⟨*U*_vdW_⟩_2_ – ⟨*U*_vdW_⟩_1_] + β­[⟨*U*_elec_⟩_2_ – ⟨*U*_elec_⟩_1_], using standard parameters α and β values equal to 0.18 and 0.50, respectively.

## 3 Results and Discussion

### 3.1 MWCNT Characterization

In this work, CS nanocomposite hydrogels containing MWCNT were used as inks for obtaining 3D-printed films for pesticide extraction. The MWCNT was initially characterized by TEM, and the obtained micrographs are presented in Figure 2.

**Figure 2:**
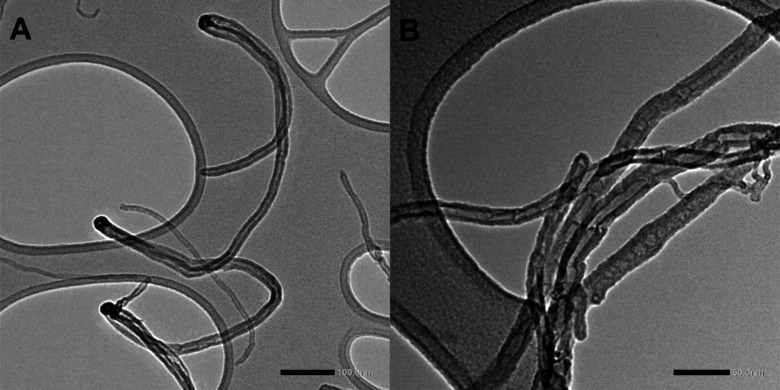
TEM images of MWCNT dispersion (acceleration voltage of 120 kV, magnification of (A) 50k× and (B) 100k×).

The images show the presence of a nanomaterial in a cylindrical shape, with a hollow channel and thick walls, thus demonstrating its tubular structure, as expected from MWCNT. The tube diameter was measured by evaluating the distance between its walls,^38^ and values between 15 and 23 nm were measured for the outer diameter (considering the outer limits of the tube) and between 4 and 7 nm for the inner diameter. It is important to emphasize that these materials possess a high surface-to-volume ratio due to their nanometric dimensions, which can be relevant for modulating the properties of the polymeric matrix such as hydrophobicity. Furthermore, they are known for their ability to extract pesticides through π-π interactions or hydrophobic interactions.^39^

### 3.2 HG-CS/MWCNT and HG-CS Characterization

CS is an important biopolymer used as an ink in 3D printing by SSE, given its appropriate viscosity in the form of a hydrogel. CS is insoluble in common solvents above its p*K*_a_ (pH = 6.5); therefore, to obtain the hydrogel ink, an acetic acid solution was used for CS solubilization. This behavior is due to the protonation of the −NH_2_ group of CS to −NH_3_^+^ in an acidic medium, which helps to increase its solubility.^40^ Considering the pH-dependent behavior of CS, adjustments in the pH of CS-based hydrogels can modulate their properties by modifying chitosan crosslinking degree^41^ and improving the hydrogel's printability. Although it has great potential for 3D printing due to its adjustable viscosity, CS exhibits hydrophilic behavior, which may limit its potential for extracting hydrophobic compounds. The addition of MWCNT in the preparation of the HG-CS/MWCNT hydrogel was carried out to provide more effective interactions with nonpolar analytes such as certain pesticides.

The obtained HG-CS and HG-CS/MWCNT hydrogels were firstly characterized by their pH and the measured values were 4.97 ± 0.02 and 4.95 ± 0.02, respectively. Evaluating the results through an unpaired *t*-test (confidence level *p* < 0.05), the pH results showed no significant difference and are therefore considered statistically equal.

In order to evaluate the printability and influence of the presence of MWCNT in the 3D printing process, the hydrogels were characterized in terms of their rheological properties. The hydrogels were evaluated when submitted to a flow ramp, assessing the behavior of their viscosities (η) and stress (τ) as a function of the shear rate (γ), and the obtained data are presented in Figure 3A.

**Figure 3:**
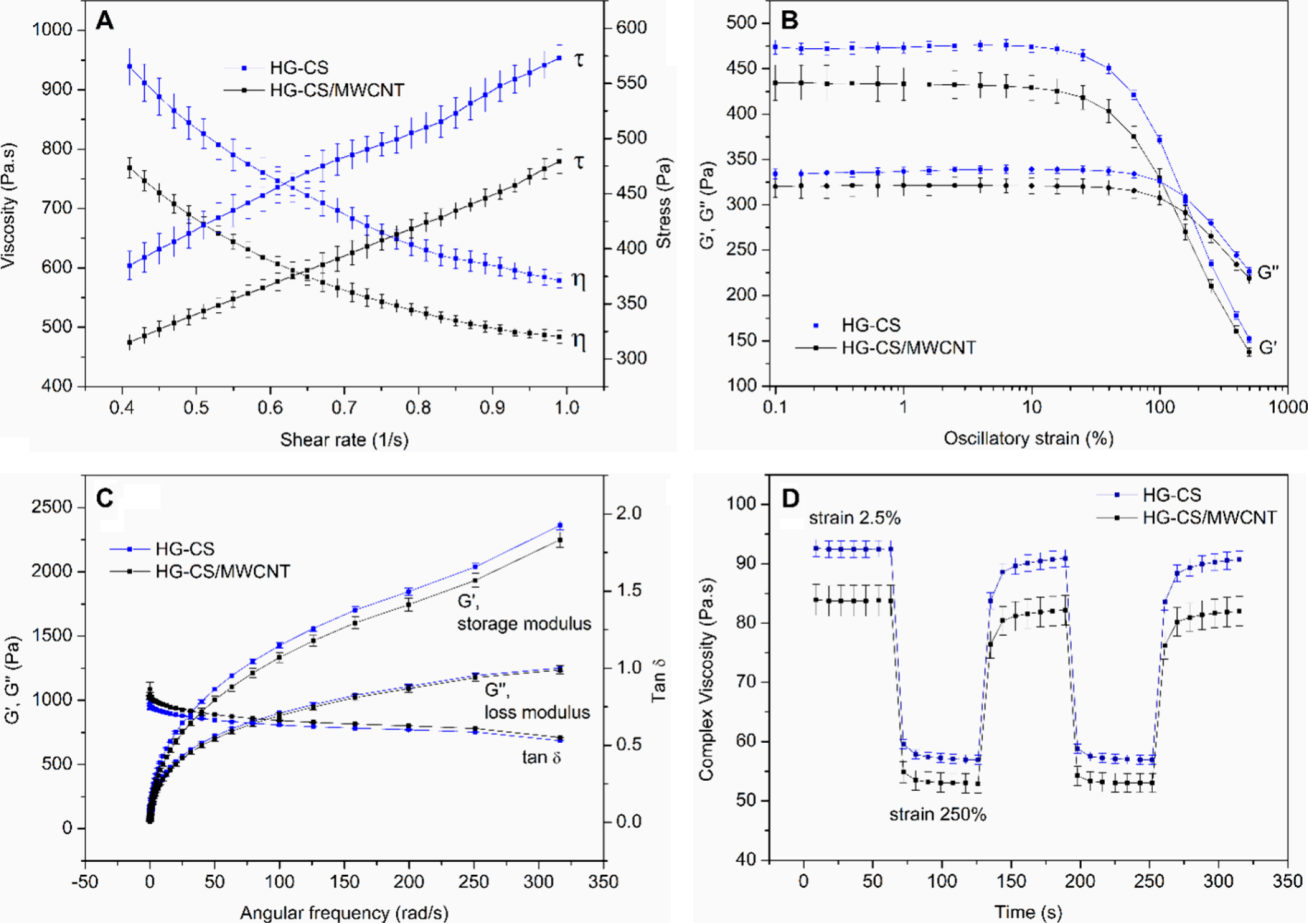
A) Flow curves showing the shear stress (τ) and apparent viscosity (η) as a function of the shear rate (γ). B) Oscillatory amplitude curves, with *G*′ (elastic or storage modulus) and *G*″ (viscous or loss modulus). C) Oscillatory frequency data showing *G*′, *G*″, and tangent delta (tan δ) as a function of angular frequency. D) Thixotropy analysis, alternating strain between 2.5 and 250 %. Values are presented as the mean ± SD (*n* = 3).

The flow curves showed a decrease in the apparent viscosity (η) with the increase in shear rate (γ), a typical pseudoplastic behavior of non-Newtonian fluids.^42^ This characteristic is desirable for the hydrogel extrusion process during 3D printing, where the material inside the syringe should not flow at rest, only when minimal stress is applied. Then, its viscosity decreases, and this allows extrusion through the syringe nozzle and deposition of the filament onto the printing bed. The hydrogels were subjected to an oscillatory amplitude test to define the viscoelastic linear region and avoid irreversible deformation during the study. The obtained curves are presented in Figure 3B. From these curves, it is possible to determine the crossover modulus, which is the intersection of the curves (*G*' and *G*''), where from this oscillatory strain, the storage modulus (*G*') decreases, and the loss modulus (*G*'') predominates. The crossover modulus represents the yield stress, indicating the minimum force required for the material to begin flowing,^43^ which is related to the force needed for the material to be extruded during 3D printing. It is observed that the values obtained for the HG-CS (311 ± 4 Pa) and HG-CS/MWCNT (300 ± 7 Pa) hydrogels do not differ statistically (*p* < 0.05), suggesting that the presence of MWCNT does not interfere with the force required to extrude the hydrogels.

The hydrogels were studied regarding the oscillatory frequency analysis, and the results are presented in Figure 3C. This measurement evaluates the variation of the *G*′ (storage) and *G*″ (loss) moduli as a function of angular frequency. It is an important measurement for better understanding the viscoelastic properties of the hydrogel, as during 3D printing by extrusion of semi-solids the hydrogel ideally should exhibit characteristics of the two moduli, flowing under the applied pressure (viscous behavior) and then recovering its viscosity (elastic behavior) once the pressure is no longer applied. In this way, its physical integrity was preserved in the printing bed. Both HG-CS and HG-CS/MWCNT hydrogels showed curves where the elastic behavior (*G*′) predominates over the viscous behavior (*G*″). Moreover, the tangent delta value (ratio between *G*′/*G*″) exhibits a linear behavior, with values below 1, characteristic of gels.^44^ The complex viscosities at 1 Hz were 91.6 ± 1.02 and 82.75 ± 2.08 Pa s for HG-CS and HG-CS/MWCNT, respectively. Although they are slightly different, it is important to highlight that the forces needed to extrude both hydrogels (yield stress) were the same.

Next, the thixotropy of the hydrogels was evaluated by subjecting them to 5 cycles of 60 s, alternating strain of 2.5 % and 250 % for each cycle (Figure 3D). This analysis is important to understand whether the material has the ability to return to its original viscosity after being subjected to strain.^45^ In 3D printing by SSE, after the hydrogel flows through a nozzle and is deposited on the printing base, it must reduce its fluid behavior and recover its viscosity so that new layers of the material can be deposited without deformation of the formerly printed material. As shown in Figure 3D, both hydrogels exhibit a decrease in complex viscosity with the increase in strain to 250 % and then nearly return to their initial state (98 % for both HG-CS and HG-CS/MWCNT) when the strain is reduced to 2.5 % again, suggesting that a proper shape fidelity of the 3D-printed objects can be achieved after semi-solid deposition in the printing table.^46^

Based on the characterizations of the HG-CS and HG-CS/MWCNT hydrogels, it can be observed that both have similar rheological properties, being non-Newtonian materials with pseudoplastic and gel-like behavior, meeting the viscoelastic and thixotropic requirements for their application as inks in 3D printing by semi-solid extrusion. The similarity in rheological properties is most likely associated with the relatively low MWCNT loading (0.1 % w/w); however, previous studies have demonstrated that such low nanotube contents are already effective for the adsorption of different target species.^21^ Conversely, increasing the MWCNT content in chitosan-based composites has been reported to promote nanofiller aggregation,^47,48^ which could adversely affect ink homogeneity, compromising printability by altering flow behavior and increasing the likelihood of nozzle clogging during the 3D printing process. In this regard, the selected formulations represent a balanced compromise between functional performance and printability, enabling the fabrication of extractor films with customized shapes by semi-solid extrusion.

### 3.3 3D-Printed Film Characterization

The 3D-printed films, TF-CS and TF-CS/MWCNT, obtained from the HG-CS and HG-CS/MWCNT hydrogels, respectively, had their dimensions predefined in the PrusaSlicer software with sizes as shown in Figure 1A, which represents their designed dimensions. Regarding the shape, the films consist of two regions: one narrower, which serves as the base and does not come into contact with the sample during extraction, and another wider region, which serves as the extracting surface. The actual dimensions of the film soon after printing were measured using a digital caliper. After printing, these films, still wet, were immediately subjected to drying, achieving an appearance and dimensions consistent with the expected ones for use as a thin film for SPME. The measured values, using a digital caliper, are shown in Table 1. Photos from the films before and after drying are shown in Figure S1 in the Supporting Information.

**Table 1:** Dimensions of the Extracting Part of 3D-Printed CS-Based TF-SPME Devices (*n* = 15)

	Length (mm)	Width (mm)
CAD design	20	5
TF-CS (wet)	20.54 ± 0.25	5.29 ± 0.15
TF-CS (dry)	20.07 ± 0.23	5.11 ± 0.14
TF-CS/MWCNT (wet)	20.28 ± 0.13	5.05 ± 0.06
TF-CS/MWCNT (dry)	20.05 ± 0.19	4.95 ± 0.06

The dimensional fidelity of the 3D-printed films was evaluated by comparing the printed dimensions with those defined in the CAD model relative to the length and width. The results are presented as boxplots in Figure 4A.

**Figure 4:**
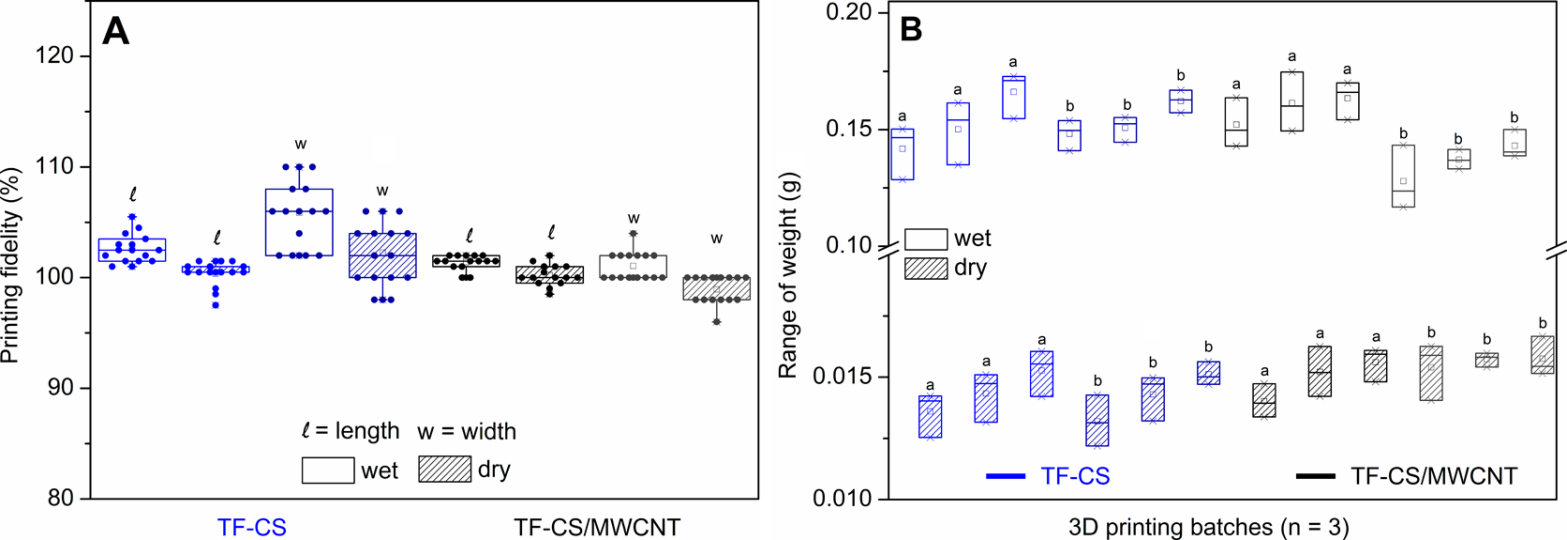
3D printing reproducibility: (A) boxplots of the printing fidelity of multiple 3D-printed films relative to the length (*l*) and width (*w*) of the CAD design (*n* = 15); (B) boxplots of the mass distribution of multiple 3D-printed films, showing intrabatch reproducibility (*n* = 3) and interbatch reproducibility (intraday and interday, where batches identified with the same letter, a or b, were printed on the same day).

The boxplots of dimensional fidelity relative to the CAD model (Figure 4A) indicate that the 3D-printed films exhibit narrow distributions for both length and width, regardless of the material (TF-CS and TF-CS/MWCNT), with fidelity values consistently close to 100%, reflecting good dimensional consistency among replicates. Slightly higher variability is observed for the width compared to the length, which can be attributed to its smaller nominal dimension (5 mm versus 20 mm for length), for which small absolute deviations result in more pronounced relative variations. Nevertheless, the absence of pronounced outliers and the overlap between the distributions indicate that these variations are systematic and well controlled and do not compromise the overall dimensional fidelity of the printed films. Moreover, a slight reduction in dimensions is observed after drying, as expected due to water evaporation.

The reproducibility of the 3D printing process was further evaluated through the mass distribution of the printed films (Figure 4B), considering both intrabatch and interbatch (intradaily and interdaily) variability. The boxplots reveal limited dispersion of mass values within individual batches, indicating good repeatability during a single printing session. Comparisons among batches printed on the same day, identified by identical letters, show similar ranges, demonstrating consistent printing performance throughout the day. Likewise, batches printed on different days do not exhibit systematic differences in mass distribution, indicating that the process maintains good interdaily reproducibility, even in the presence of potential environmental or operational variations. Soon after printing, the average mass of the wet films was 0.15322 ± 0.01164 g for TF-CS and 0.14751 ± 0.01553 g for TF-CS/MWCNT (*n* = 18).

The loss on drying test showed losses of 90.66 ± 0.36 % for TF-CS and 89.54 ± 1.2 % for TF-CS/MWCNT (*n* = 18), consistent with the solvent content used in the hydrogel preparation. The dry films weighted 14.30 ± 1.08 mg and 15.29 ± 0.93 mg for TF-CS and TF-CS/MWCNT, respectively. After NaOH treatment, the average mass of the TF-CS films was 11.04 ± 0.81 mg (RSD 7.34%), and for TF-CS/MWCNT, it was 12.43 ± 0.51 mg (RSD = 4.1 %). It can be observed that there was a difference between the average masses, with TF-CS/MWCNT films showing a higher mass compared to TF-CS films (independent two-sample *t*-test, *p* < 0.0001). Both films showed masses around 10 mg, but the TF-CS films exhibited greater variability, leading to a higher RSD value. Since the films have masses in the milligram range, subtle variations during the 3D printing process can have an influence on the reproducibility of such miniaturized extractor devices, since the final dimensions of the films after NaOH treatment (dry) were *ca*. 15 mm × 4 mm. On the other hand, this technique holds great potential for obtaining modified materials, given the ease of incorporating species of interest, such as MWCNT, into the polymeric matrix.

The 3D-printed CS-based films were characterized by SEM, to evaluate the incorporation and dispersion of the MWCNT into the polymeric matrix, and the acquired images are presented in Figure 5. As observed in Figure 5A, the TF-CS film (without MWCNT) exhibits a relatively homogeneous and continuous morphology, which is characteristic of the neat polymer matrix. In contrast, Figure 5B clearly reveals the presence of the MWCNT embedded within the chitosan matrix. The nanotubes appear to be homogeneously dispersed, with no evidence of large agglomerates or phase-separated domains at the observed scale.

**Figure 5:**
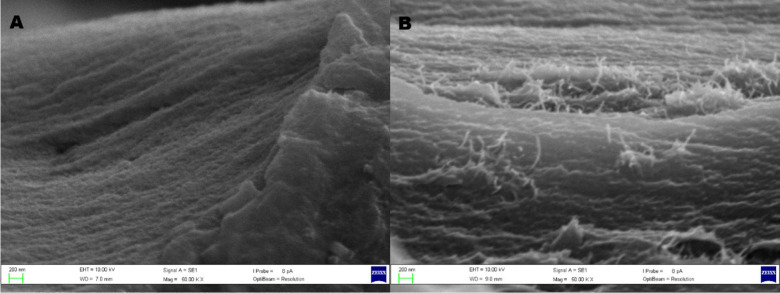
SEM images of 3D-printed CS-based films: (A) TF-CS and (B) TF-CS/MWCNT (acceleration voltage of 10 kV and magnification of 50k×).

Since the films were designed for use as extracting phases for pesticides from aqueous samples, followed by desorption of the analytes in acetonitrile, the films were studied for their swelling behavior in water and deswelling in acetonitrile. The swelling in water was evaluated in the films before and after being subjected to a NaOH solution bath, with the aim of assessing the impact of the process on the film's hydrolytic stability. The swelling curves are presented in Figure 6A below.

**Figure 6:**
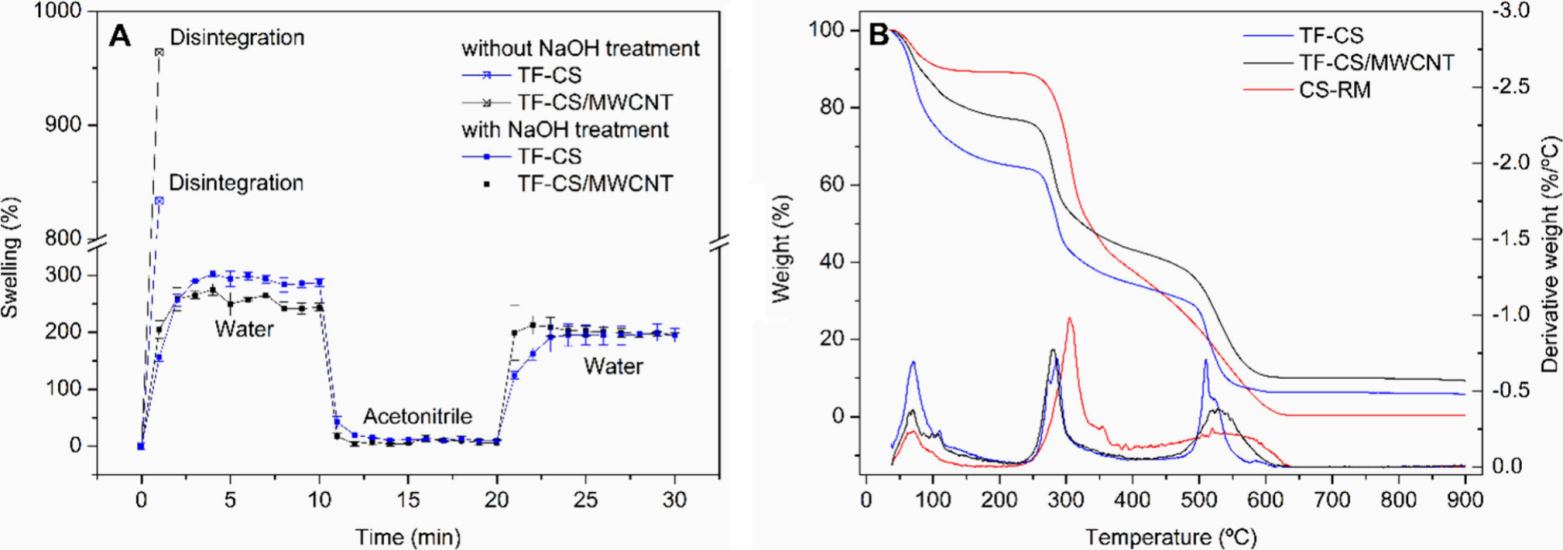
A) Swelling percentage as a function of time for TF-CS/MWCNT and TF-CS before and after treatment with NaOH solution. B) Thermogravimetric analysis data for TF-CS/MWCNT and TF-CS are compared to CS-RM.

It is possible to observe that the NaOH solution treatment impacts the hydrolytic stability of the films, as the untreated films disintegrated after the first minute of contact with water. In contrast, the treated films exhibited swelling of 244 ± 7% in water within 10 min for TF-CS/MWCNT and 287 ± 7% for TF-CS under the same conditions, both remaining intact throughout the experiment. The swelling degree of TF-CS was significantly higher than that of TF-CS/MWCNT (independent two-sample *t*-test, *p* < 0.01), and this can be attributed to the incorporation of MWCNT, which may slightly increase the hydrophobic character of the films even at low loadings. However, this effect does not significantly alter the overall hydrophilic nature of the materials, as reflected by the swelling behavior, since the macroscale surface properties remain predominantly governed by the chitosan matrix. After 10 min, the TF-CS and TF-CS/MWCNT films were immersed in acetonitrile, where it was observed that both returned to mass values close to the initial ones (only 5% higher than the initial mass for TF-CS/MWCNT and 9% for TF-CS). When re-exposed to water, a swelling of 195% was observed for both TF-CS/MWCNT and TF-CS, with the material remaining intact for additional 10 min. In this way, the treatment with a 1 mol L^–1^ NaOH solution plays an important role in the hydrolytic stability and control of the swelling level of the films in water and acetonitrile. This result is consistent with other studies demonstrating the impact of NaOH solutions on CS crystallinity.^41^ These parameters are crucial for maintaining the integrity of the extraction phase in the solvents used in the pesticide extraction process from aqueous samples.

The thermal behavior of the 3D-printed films and the chitosan raw-material (CS-RM) was evaluated by thermogravimetric analysis, and the results are presented in Figure 6B. The films presented 20–30% of residual moisture, as expected for polysaccharides due to their affinity for water and easy hydration.^49^ The thermo-oxidative degradation of chitosan in all samples occurred in two stages, 250–350 °C and 470–630 °C, in agreement with previous findings.^50^ A shift of ca. 30 °C in the degradation temperature of the first stage was observed when comparing the 3D-printed films with CS-RM, suggesting a lower thermal resistance of CS in the printed structures. This behavior is in line with previous reports, where at low degrees of cross-linking, the formation of intra-cross-linking reactions between polysaccharides chains may interfere in pre-existing hydrogen bonding interactions, weakening the polymer structure and decreasing thermal stability.^49^ Nevertheless, both the 3D printing process and the microextraction application are carried out under ambient conditions, meaning that this minor reduction in thermal stability has no adverse effect on the practical performance of the material in the proposed analytical context.

### 3.4 3D-Printed TF-CS and TF-CS/MWCNT Extraction Evaluation

The 3D-printed films, TF-CS and TF-CS/MWCNT, were studied as extracting materials for 26 analytes composed of organochlorine and organophosphorus pesticides. CS, as a polymeric matrix, has −OH and −NH_2_ groups available for interacting with analytes, while MWCNTs are known for their potential to extract organic compounds through hydrophobic interactions or π–π interactions with aromatic rings.^51^ The extraction assays were performed in triplicate, and the extraction potentials of the TF-CS and TF-CS/MWCNT films for the analytes are presented in Figure 7. Representative chromatograms are shown in Figure S2 (Suppf orting Information).

**Figure 7:**
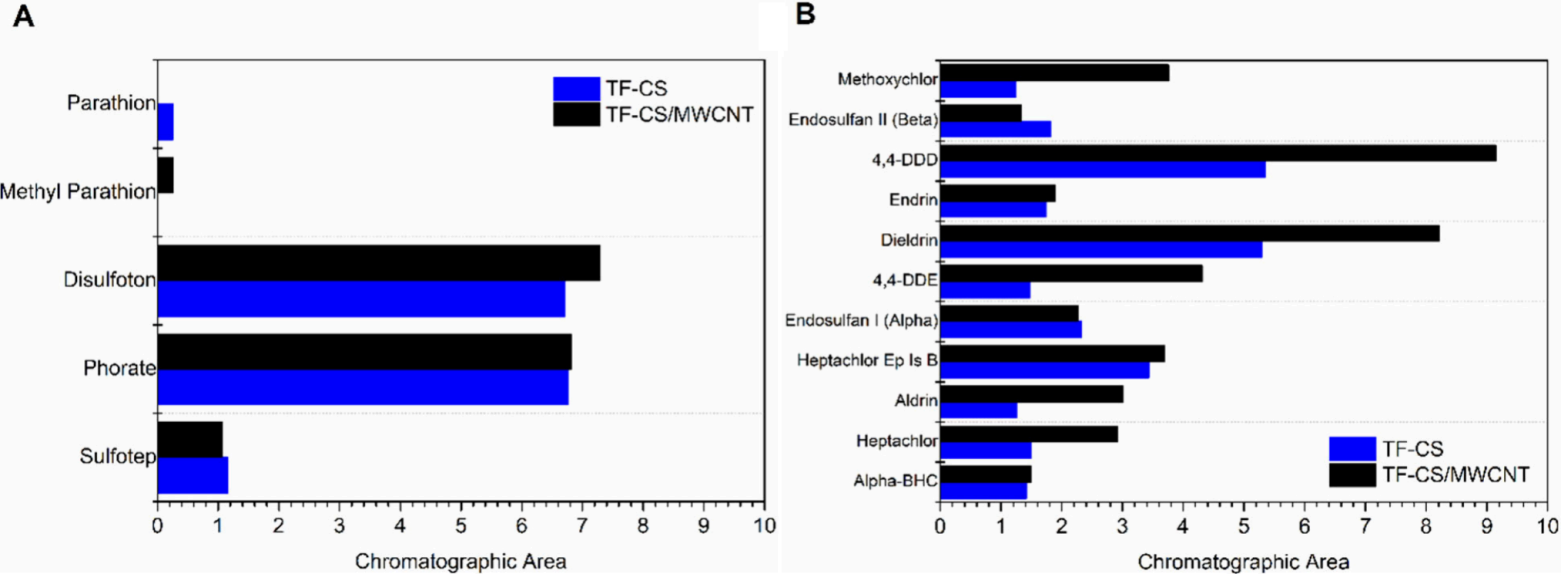
Normalized chromatographic area values (analyte/IS ratio) for each organophosphorus (A) and organochlorine (B) compound identified in the analysis.

It can be observed that the materials extracted 5 organophosphorus and 11 organochlorine pesticides, with the TF-CS/MWCNT film demonstrating better extraction performance compared to TF-CS for 3 and 9 analytes, respectively. Notably, all pesticides for which the TF-CS/MWCNT film exhibited a markedly improved extraction efficiency over the TF-CS film had log *P* values greater than 5.0: methoxychlor (5.1), 4,4-DDD (6.0), 4,4-DDE (6.5), aldrin (7.4), heptachlor (5.4), and dieldrin (6.2). The remaining analytes, parathion (3.8), methyl parathion (3.0), disulfoton (4.0), phorate (3.9), sulfotep (4.0), endosulfan II (3.8), endrin (5.3), endosulfan I (3.8), heptachlor epoxide isomer B (5.4), and alpha-BHC (3.7), were extracted more similarly by both materials. This could suggest that the incorporation of the MWCNT enhances the extraction potential for more hydrophobic compounds. MWCNT are known for their ability in extracting organic and nonpolar compounds via hydrophobic interactions and π–π stacking with aromatic rings.^51–53^ Moreover, the TF-CS film also demonstrated an extraction performance, reinforcing the potential of chitosan as a versatile matrix for microextraction. Its combination with MWCNT further enhances the tunability of the extraction phase, enabling the tailoring of chemical affinities to target specific classes of analytes. This synergy between the polymer and nanomaterial suggests a promising platform for selective and customizable microextraction.

Molecular dynamics (MD) simulations were performed to provide mechanistic insight into the enhanced extraction behavior of the CS/MWCNT system using 4,4-DDD as a representative hydrophobic organochlorine pesticide. Analysis of the final 25 ns of the simulations showed that, in the CS-only system, 4,4-DDD remained tightly associated with the CS matrix, exhibiting persistent close approach, extensive contacts, limited solvent exposure, and restricted mobility, consistent with partitioning into a CS-rich microenvironment (Table S2).

In the CS/CNT system, no direct adsorption of 4,4-DDD onto the nanotube surface was observed (Figure 8). Instead, the nanotube remained predominantly coated by CS, as indicated by the strongly favorable CS:CNT van der Waals term ⟨LJ­(SR⟩ = −3260.8 kJ mol^–1^). A Linear Interaction Energy (LIE) analysis (Table S3) yielded a negative ΔΔ*G* value (∼−6.6 kJ mol^–1^), suggesting a more stabilizing microenvironment for the analyte upon incorporation of CNT. Taken together, these results indicate that the role of CNT could be primarily indirect, promoting microstructural and hydration changes in the CS matrix rather than acting as a direct adsorption site for 4,4-DDD. Nonetheless, further studies are necessary to better elucidate the mechanisms that favored the extraction of specific pesticides in each material, e.g., analyte’s chemical affinity for the extraction phase, analyte’s diffusion kinetics through the polymeric matrix for all analytes, and competitive adsorption phenomena when multiple analytes are present.

**Figure 8:**
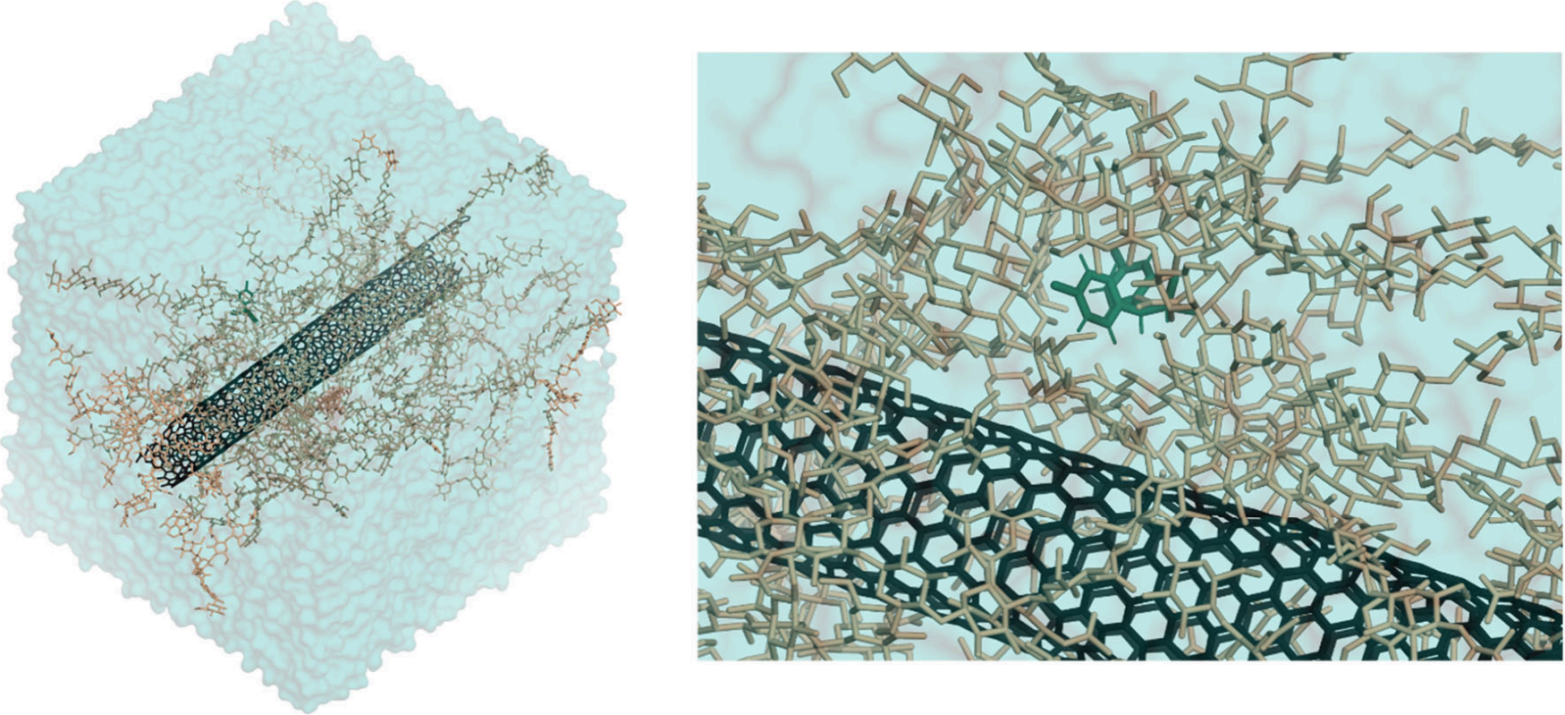
Representative molecular dynamics models of the CS/CNT (CS in beige, CNT in black) in the presence of 4,4-DDD (in green), used to investigate analyte-sorbent interactions in an aqueous environment (water in cyan).

Due to the better extraction performance, the TF-CS/MWCNT material was used for the optimization of the methodology, employing a Doehlert design with 5 and 3 levels for the extraction time and NaCl content parameters (Supporting Information, Table S4), aiming to enhance the extraction process. The generated statistical model was significant, presenting *r* ^2^ = 0.95436, which is an acceptable value for the model. Moreover, the random dispersion of the residues and the obtained responses demonstrated no lack of fit. The results showed that higher values of extraction time and NaCl concentration resulted in greater extraction (response surface obtained is shown in Figure S3 in the Supporting Information); however, it was observed that an extraction time of 120 min and 30% NaCl (w/v) already provided adequate and satisfactory responses for the intended application. Therefore, to develop a feasible and less time-consuming process, the conditions of 120 min of extraction and 30 % NaCl were established for the extraction in this work.

To evaluate whether there is a linear extraction response for the analytes, the TF-CS/MWCNT material was tested under the optimized conditions with samples of different concentrations of the analyte mix. Among the 26 analytes evaluated, 7 showed a linear extraction response with the TF-CS/MWCNT material within the assessed concentration range, as shown in Table 2. Linearity was evaluated considering an *r* ^2^ value greater than or equal to 0.990, according to the international AOAC guidelines.^54^ The linearity graphs are shown in Figure S4 in the Supporting Information.

**Table 2:** Linear Range, Linear Equation, Coefficient of Determination, and LOQ for Seven Pesticides

Analyte	Linear range (μg L^–1^)	Linear Equation	*r* ^2^	LOQ (μg L^–1^)
Alpha-BHC	50–500	*y* = 1.25*x* – 21.63	0.9951	50
Aldrin	100–700	*y* = 3.60*x* – 406.38	0.9929	100
Endosulfan I (alpha)	50–700	*y* = 2.01*x* – 64.53	0.9913	50
Dieldrin	100–700	*y* = 9.91*x* – 919.95	0.9979	100
4,4-DDE	200–700	*y* = 10.04*x* – 1287.91	0.9900	200
4,4-DDD	100–700	*y* = 11.80*x* – 1181.13	0.9975	100
Phorate	50–500	*y* = 6.40*x* – 376.88	0.9912	50

The relative standard deviation (RSD, *n* = 3) of the chromatographic response was calculated at each concentration level for all analytes that exhibited linearity. For the lowest concentration (50 μg L^–1^), RSDs ranged from 0.4% to 15.3%; at intermediate level (400 μg L^–1^), values ranged from 9.2% to 19.3%; and at the highest concentration (700 μg L^–1^), RSDs were between 0.8% and 20.1%. The observed variability for some analytes can be mainly attributed to small variations in the printing process, as well as to film swelling behavior and differences in analyte–sorbent interactions that influence desorption efficiency. All values were within the acceptable range established by AOAC international guidelines, which recommend a maximum RSD of 22% for assessing reproducibility at concentrations of around 100 μg L^–1^.^54^ Furthermore, the LOQ was determined as the first concentration within the linear range, resulting in a LOQ of 50 μg L^–1^ for the compounds alpha-BHC, endosulfan I (alpha), and phorate and 100 μg L^–1^ for aldrin, dieldrin, and 4,4-DDD; and 200 μg L^–1^ for 4,4-DDE.

Among the pesticides for which a linear response was identified, 6 are organochlorines (alpha-BHC, aldrin, endosulfan I (alpha), dieldrin, 4,4-DDE, and 4,4-DDD) and only 1 is an organophosphorus pesticide (phorate). A hypothesis for this behavior is that the detected organochlorine pesticides have a predominantly nonpolar nature, which likely favors interactions with the MWCNT-containing extraction phase. On the other hand, organophosphorus compounds tend to be more polar, which could have, in comparison, weaker interactions with the MWCNT-containing extracting material. It is important to note that adsorption onto CNTs is governed by multiple mechanisms, including hydrophobic forces, π–π stacking, hydrogen bonding, and electrostatic interactions, each of which can be influenced by the surrounding medium.^53^ In aqueous systems, hydrophobic effects tend to dominate, often overshadowing other interactions such as hydrogen bonding, particularly when unmodified CNTs are used. As a result, nonpolar analytes are preferentially extracted under these conditions. Nevertheless, the presence of −NH_2_ and −OH groups in CS introduces polar interaction sites and can contribute to the extraction of more polar compounds through hydrogen bonding and van der Waals interactions, broadening the material’s selectivity.

A comparative overview of the analytical performance of 3D-printed TF-CS/MWCNT and other 3D-printed SPME devices reported in the literature is presented in Table 3, highlighting printing techniques, adsorbent materials, target analytes, and limits of quantification (LOQ).

**Table 3:** Comparative Analysis of the Analytical Features Obtained in This Study and Other Reported SPME 3D-Printed Materials

Adsorbent	Shape and application	3D printing technique	Analytes	LOQ (μg L^–1^)	Ref
Polyamides (PA6 and PA12) reinforced with 15% of carbon fiber	TF-SPME	FDM	38 small molecules with log P range of 0.2–7.2 (THC, cocaine, clonazepam, etc.)	1–5	^5^
Polypropylene/acrylonitrile-butadiene-styrene/C18-functionalized silica	Honeycomb-like and tubular shape for SPME	FDM	3 low-molecular-weight analytes (glimipiride, imipramine, and carbamazepine)	N/A	^55^
Polyamide reinforced with 15% of carbon fiber	Honeycomb-like disk for rotating-disk sorptive extraction	FDM	20 organochlorine and organophosphorus pesticides	0.5–10	^22^
Polymeric ionic liquid	Blade-type fibers for TF-SPME	LCD	10 organic contaminants (plasticizers, antimicrobial agents, UV filters, and pesticides)	0.43–150	^6^
Photo-curable resin and C18-modified silica	Disk-shaped and volumetric lattice 3D devices for SPE	DLP	2 drugs (diazepam and medazepam)	50	^56^
CS/MWCNT	TF-SPME	SSE	7 organochlorine and organophosphorus pesticides	50–200	This work

As shown in Table 3, the extraction performance achieved in this study is comparable to other 3D-printed microextraction systems reported in the literature. A key distinguishing feature is the composition of the extraction phase: while previous works mostly employed synthetic polymers or petrochemical-based materials, this study utilized chitosan, a natural, biodegradable, and renewable biopolymer, reinforcing the alignment with green chemistry principles. Additionally, unlike other studies that used FDM or light-based printing techniques, the present work explored SSE 3D printing. This approach facilitated the incorporation of MWCNT into the printable ink, which enhanced the extraction performance.

When the TF-CS/MWCNT is compared with other non-3D-printed TF extraction devices, several approaches reported in the literature can be highlighted. da Paz Braga et al. (2025) developed a method for the determination of multiple pesticides in paddy water using polymer powders (Strata XA-W and Strata FL-PR) placed onto double-sided adhesive tape, achieving LOQ in the range of 0.05 and 10 μg L^–1^.^57^ Jabali et al. (2019) employed commercially available SPME fibers based on polyacrylate (PA) and polydimethylsiloxane (PDMS) for the simultaneous analysis of 48 pesticides, obtaining LOQ ranging from 0.04 to 1.43 μg L^–1^ for PA fiber under optimized conditions.^58^ In another approach, Valenzuela et al. (2020) reported the deposition of carbon nanomaterials onto steel threads by chemical vapor deposition, which were applied to the microextraction of 24 pesticides from water, yielding LOQ in the range 0.0007–3.7320 μg L^–1^.^59^ Although the LOQ obtained in this work is higher than those reported for some TF extraction approaches, the SSE-based fabrication avoids multistep procedures, enabling the direct fabrication of the extraction films in their final geometry, without molds. Beyond the low cost and accessibility, this approach also enables the production of multiple extraction devices, supporting high-throughput workflows in routine analytical applications.

The stability of the films was evaluated by storing the samples for 21 days and comparing their performance with that of a freshly prepared batch, with extractions carried out under identical conditions. The responses obtained for the analytes are shown in Figure S5. No statistically significant differences were observed between Day 1 and Day 21 for six of the seven analytes (paired *t*-test: *p* = 0.070 for alpha-BHC, *p* = 0.215 for aldrin, *p* = 0.134 for dieldrin, *p* = 0.109 for 4,4-DDE, *p* = 0.112 for 4,4-DDD, and *p* = 0.585 for phorate). A statistically significant difference was observed only for endosulfan I (*p* = 0.034). Additionally, when results from Day 1 and Day 21 were pooled and evaluated as a single batch, the RSD values ranged from 14% to 23% across the analytes (16% for alpha BHC, 23% for aldrin, 23% for endosulfan I, 17% for dieldrin, 21% for 4,4-DDE, 18% for 4,4-DDD, and 14% for phorate). Overall, the RSD values were within or close to the 20% acceptance criterion recommended by AOAC International guidelines.^54^

The reusability of the extraction films was preliminarily evaluated by reusing a single film and comparing its response to films from the same batch at the same analyte concentration. The results, expressed as relative response percentages, indicate analyte-dependent behavior. While alpha-BHC retained a high response level (88.2 %) after reuse, the remaining analytes exhibited a pronounced decrease in response, ranging from 15.6% to 39.1%. These results suggest that the extraction films present limited reusability under the evaluated conditions, likely due to partial saturation of adsorption sites or incomplete desorption of strongly retained analytes. Therefore, the films are more suitable for single-use applications. It is important to note that the films are fabricated from renewable, low-cost materials and can be produced by rapid and flexible prototyping using 3D printing. Moreover, single-use materials eliminate the memory (carryover) effect, which arises when analytes are not completely desorbed and subsequently contaminate following extraction cycles.

To evaluate the applicability of the proposed method, two lake water samples collected in the metropolitan region of Porto Alegre, Brazil, identified as PA and PB, were analyzed. The chromatograms obtained for the real samples are presented in Figure S6. No target analytes were detected in *in natura* samples (PA-1, PA-2, PA-4, PB-1, PB-2, and PB-4), with concentrations below the method detection limits. In contrast, for the spiked samples (PA-3 and PB-3), all seven analytes were successfully identified in the chromatograms, demonstrating the potential of the proposed method for the analysis of complex aqueous matrixes.

## 4 Conclusions

A film fabricated via SSE 3D printing, based on CS and MWCNT, proved to be a promising device for the extraction of seven pesticides (alpha-BHC, aldrin, endosulfan I (alpha), dieldrin, 4,4-DDE, 4,4-DDD, and phorate) for application in TF-SPME, followed by determination using GC-MS. The CS hydrogel-forming ability enabled the preparation of a hydrogel under optimal printing conditions, exhibiting appropriate viscosity at rest, flowing when subjected to an applied force, and recovering its original characteristics after deformation, as demonstrated by the assessment of rheological parameters. The printed films showed good dimensional and mass reproducibility as well as adequate stability in both aqueous media and acetonitrile, maintaining its structural integrity throughout the extraction process. The printed CS/MWCNT nanocomposite demonstrated the ability to concentrate the analytes and act as an extractive phase for TF-SPME. Molecular dynamics simulations provided insights into the extraction process, indicating that the incorporation of the MWCNT could promote microstructural and hydration effects within the chitosan matrix that favor analyte-sorbent interactions. Seven pesticides (alpha-BHC, aldrin, endosulfan I, dieldrin, 4,4-DDE, 4,4-DDD, and phorate) were extracted with linear responses within the evaluated concentration range and limits of quantification between 50 and 200 μg L^–1^. Storage stability experiments with real environmental water samples provided an initial assessment of the applicability of the proposed device. In addition to its analytical performance, the devices were based on a renewable matrix and were processed by an SSE printing technique under environmentally friendly conditions, holding a promising future for the integration of 3D printing, sample preparation, and green chemistry.

## Supplementary Material



## Notes

Table of quantification ion, identification ion, retention time and chemical structure of the identified analytes and IS, photos of moist films dimensions, chromatograms, table of structural and dynamical descriptors, table of interaction energies, table of Doehlert matrix, response surface, linearity graphs, and storage stability and reusability (PDF)
