# Supplementary material for: Semi-solid Extrusion 3D Printing of Chitosan/Carbon Nanotube Nanocomposite Films for Microextraction of Pesticides in Water
Source: ACS Omega. 2026 Apr 13;11(16):23962–75. doi: 10.1021/acsomega.5c11817 (PMC13130108; doi:10.1021/acsomega.5c11817)
Supplement: Supplementary file 1 [file ao5c11817_si_001.pdf]

**Supplementary Material**

# Semi-Solid Extrusion 3D Printing of Chitosan/Carbon Nanotube Nanocomposite Films for Microextraction of Pesticides in Water

*Giuseppe da Silva Salvador[a], Vítor Augusto Bauer[b], Rita de Cássia dos Reis Schmidt[a], Nadine Lysyk Funk[c], Sofia Aquino Monteiro[a], Camila Scheid[a], Silvio Buchner[d], Cesar Liberato Petzhold[e], Ketherin Adam Antoni[a], Tiago Espinosa de Oliveira[a], Josias Merib[a]*, Monique Deon[a]**

[a] Universidade Federal de Ciências da Saúde de Porto Alegre, Programa de Pós-Graduação em Biociências, Rua Sarmento Leite 245, CEP 90050-170, Porto Alegre, RS, Brazil;

[b] Universidade Federal de Ciências da Saúde de Porto Alegre, Curso de Graduação em Química Medicinal, Rua Sarmento Leite 245, CEP 90050-170, Porto Alegre, RS, Brazil;

[c] Universidade Federal do Rio Grande do Sul, Programa de Pós-Graduação em Ciências Farmacêuticas, Av. Ipiranga, 2752, CEP 90610-000, Porto Alegre, RS, Brazil.

[d] Universidade Federal do Rio Grande do Sul, Instituto de Física, CP 15051, CEP 91501-970, Porto Alegre, RS, Brazil.

[e] Universidade Federal do Rio Grande do Sul, Instituto de Química, CP 15003, CEP 91501-970, Porto Alegre, RS, Brazil.

Table S1. Quantification ion, identification ion, retention time and chemical structure of the identified analytes and IS.

| Analyte | Chemical structure | Retention time (min) | Quantification ion (m/z) | Identification ion (m/z) |
| --- | --- | --- | --- | --- |
| 2-fluorbiphenyl (IS) | 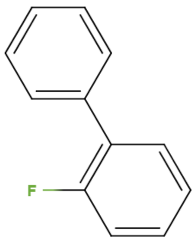 | 4.10 | 172 | 171, 170, 173 |
| Sulfotep | 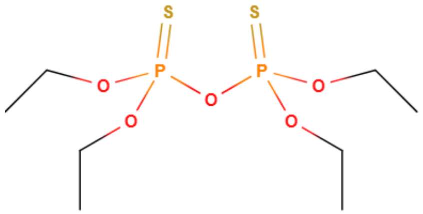 | 5.92 | 322 | 97, 202, 29 |
| Phorate | 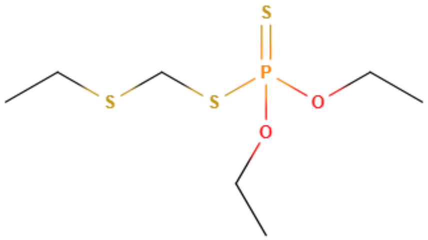 | 5.98 | 75 | 121, 29, 97 |

| | | | | |
|---|---|---|---|---|
| Disulfoton | 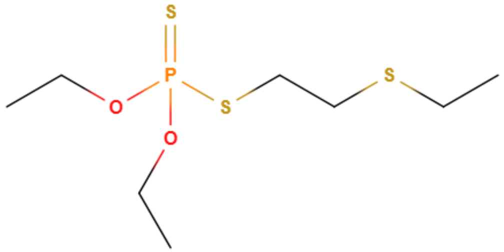 | 6.91 | 88 | 89, 97, 60 |
| Methyl Parathion | 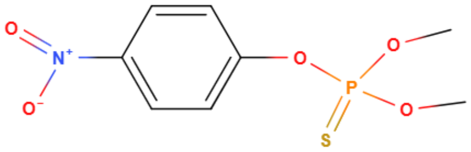 | 7.77 | 109 | 263, 125, 79 |
| Parathion | 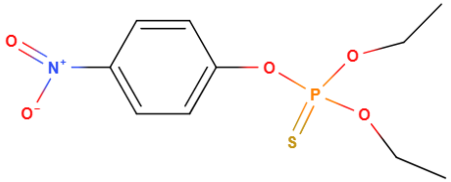 | 8.72 | 291 | 109, 97, 139 |
| Alpha-BHC | 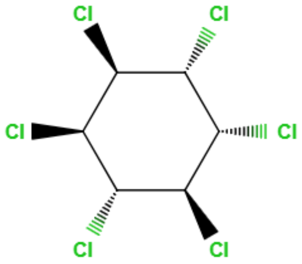 | 6.06 | 183 | 181, 219, 217 |

| | | | | |
|---|---|---|---|---|
| Heptachlor | 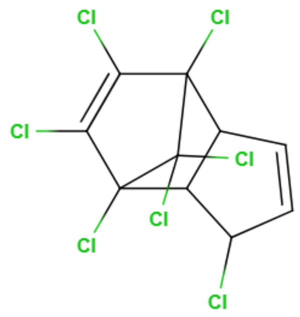 | 7.78 | 100 | 272, 274, 270 |
| Aldrin | 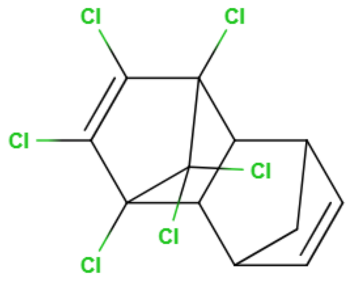 | 8.47 | 66 | 263, 265, 261 |
| Heptachlor Epoxide Isomer B | 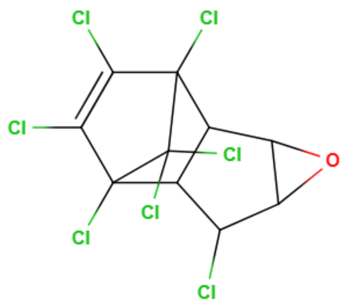 | 9.37 | 81 | 353, 355, 351 |

| | | | | |
|---|---|---|---|---|
| Endosulfan I (Alpha) | 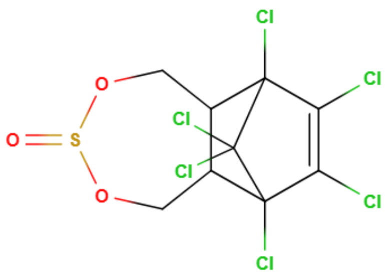 | 10.24 | 241 | 239, 195, 237 |
| 4,4-DDE | 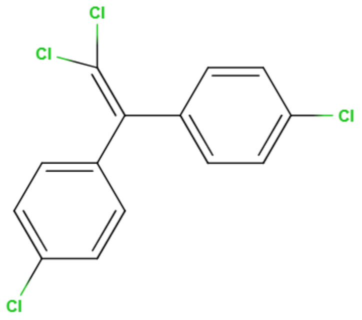 | 10.94 | 246 | 318, 248, 316 |
| Dieldrin | 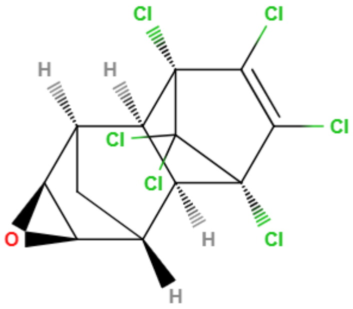 | 10.91 | 79 | 81, 82, 77 |

| | | | | |
|---|---|---|---|---|
| Endrin | 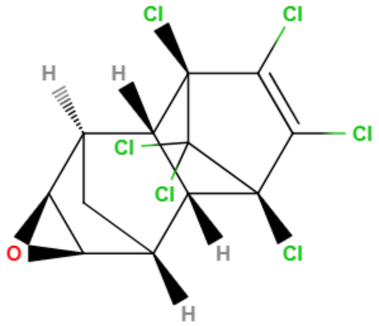 | 11.46 | 81 | 79, 263, 67 |
| 4,4-DDD | 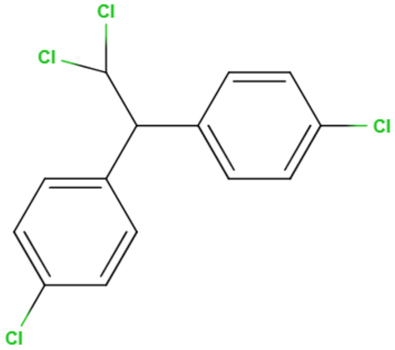 | 12.11 | 235 | 237, 165, 236 |
| Endosulfan II (Beta) | 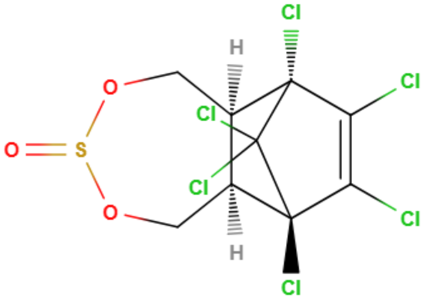 | 11.74 | 195 | 241, 237, 239 |

| | | | | |
|---|---|---|---|---|
| Methoxychlor | 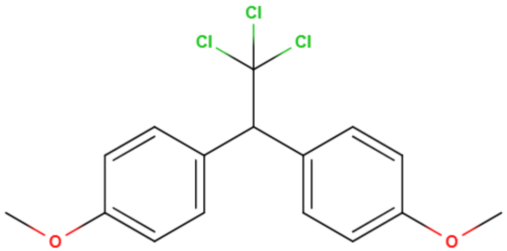 | 15.02 | 227 | 228, 212, 196 |

Figure S1. Moist films dimensions: length of fixing part (A) and extracting surface (B), width of fixing part (C) and extracting surface (D). Dried films dimensions: length of fixing part (E) and extracting surface (F), width of fixing part (G) and extracting surface (H).

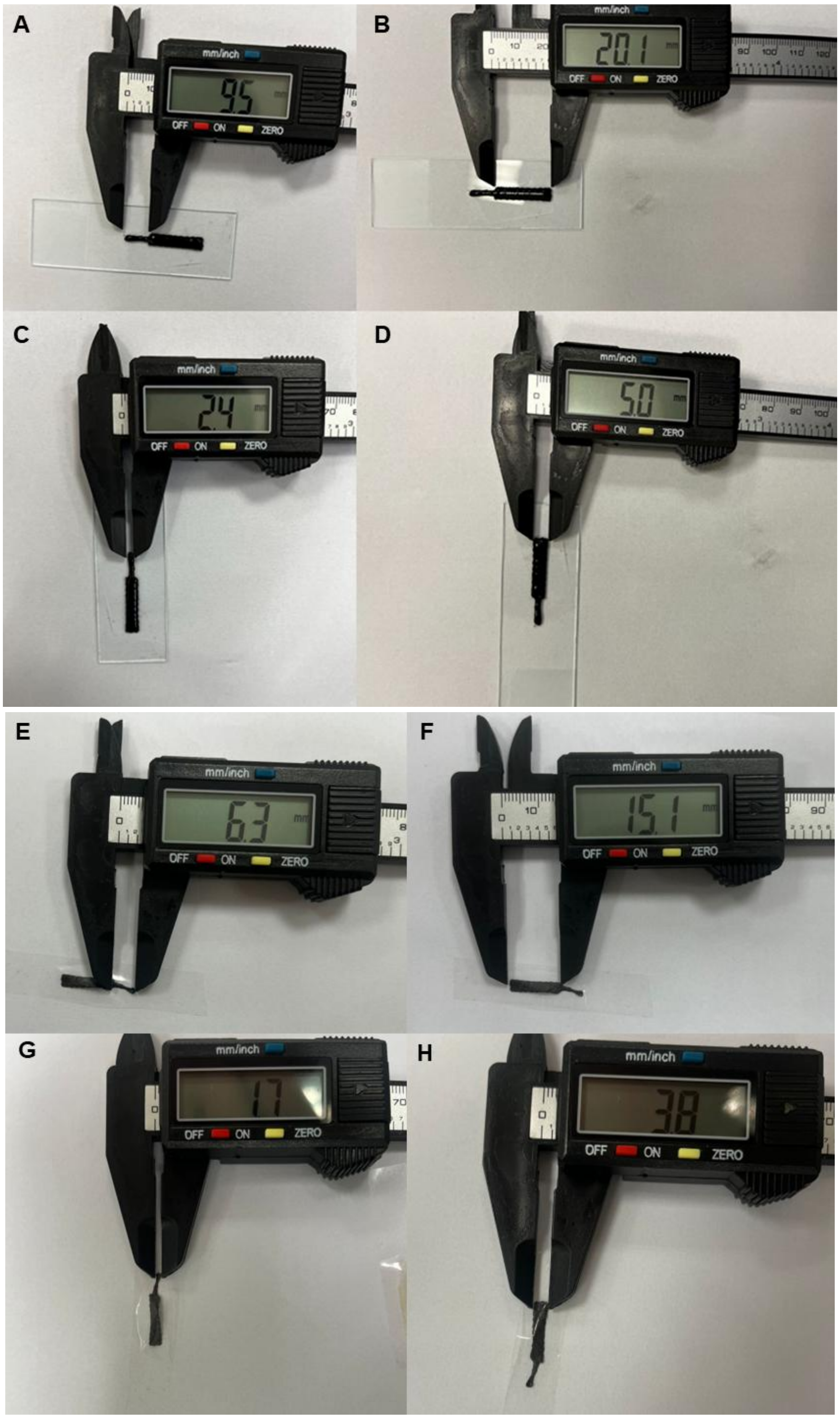

Figure S2. Alpha-BHC, 4,4-DDD, Dieldrin, 4,4-DDE, Endosulfan I (alpha), Aldrin and Phorate chromatograms at a concentration of 500 μg $L^{-1}$, and 2-fluorbiphenyl (IS) chromatogram at a concentration of 200 μg $L^{-1}$.

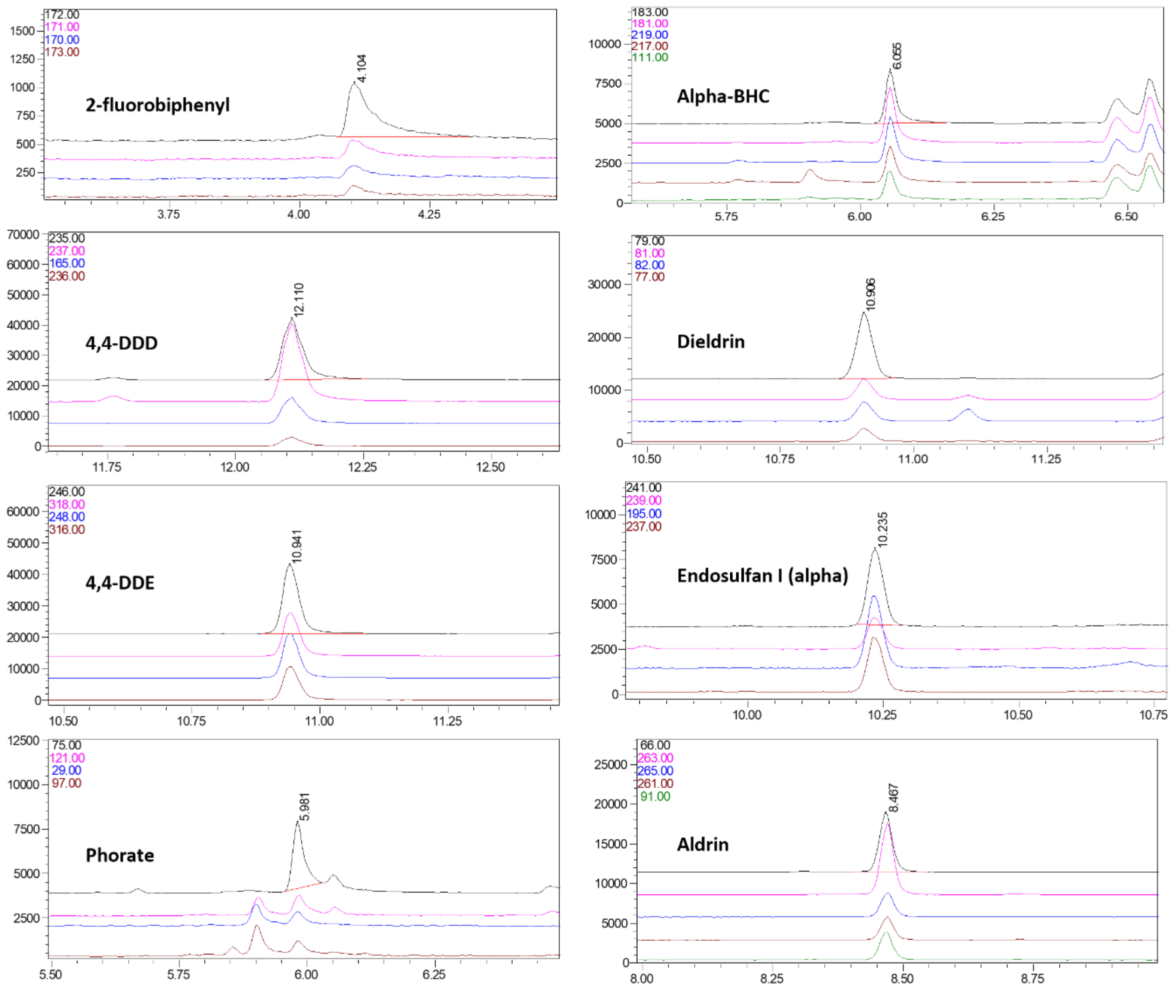

Table S2. Structural and dynamical descriptors for 4,4-DDD in CS and CS/CNT.

| **Descriptor** | **CS** | **CS/CNT** |
|---|---|---|
| ‹mindist›$_{4,4\text{-DDD:CS}}$ (nm) | 0.217 ± 0.016 | 0.207 ± 0.011 |
| ‹mindist›$_{4,4\text{-DDD:CNT}}$ (nm) | - | 0.678 ± 0.021 |
| ‹number of contacts›$_{4,4\text{-DDD:CS}}$ (nm) | 210 ± 32 | 235 ± 10 |
| SASA$_{4,4\text{-DDD}}$ (nm$^2$) | 4.88 ± 0.19 | 4.81 ± 0.15 |
| Coordination number$_{\text{Water:4,4-DDD}}$ (r = 0.6 nm) | 9.15 | 6.00 |
| MSD$_{4,4\text{-DDD}}$ (cm$^2$s$^{-1}$) | 0.0030 ×10$^{-5}$ | 0.0004 ×10$^{-5}$ |

Table S3. Short-range interaction energies for 4,4-DDD in CS and CS/CNT. All energies are in kJ mol$^{-1}$. Coul(SR) and LJ(SR) correspond to short-range electrostatic and van der Waals terms, respectively.

| **System** | **Pair** | **‹Coul(SR)›** | **‹LJ(SR)›** |
|---|---|---|---|
| CS | 4,4-DDD:CS | -34.80 | -98.69 |
| | 4,4-DDD:Water | -164.98 | -30.98 |
| CS/CNT | 4,4-DDD:CS | -125.13 | -107.52 |
| | 4,4-DDD:Water | -84.77 | -31.10 |
| | 4,4-DDD:CNT | 0.00 | -0.66 |

Table S4. Doehlert matrix.

| Experiment | Extraction Time (min) | NaCl concentration (% (w/w)) |
|---|---|---|
| 1 | 60 | 30 |
| 2 | 120 | 30 |
| 3 | 30 | 15 |
| 4.1 | 90 | 15 |
| 4.2 | 90 | 15 |
| 4.3 | 90 | 15 |
| 5 | 150 | 15 |
| 6 | 60 | 0 |
| 7 | 120 | 0 |

Figure S3. Response surface obtained for the optimization extraction conditions.

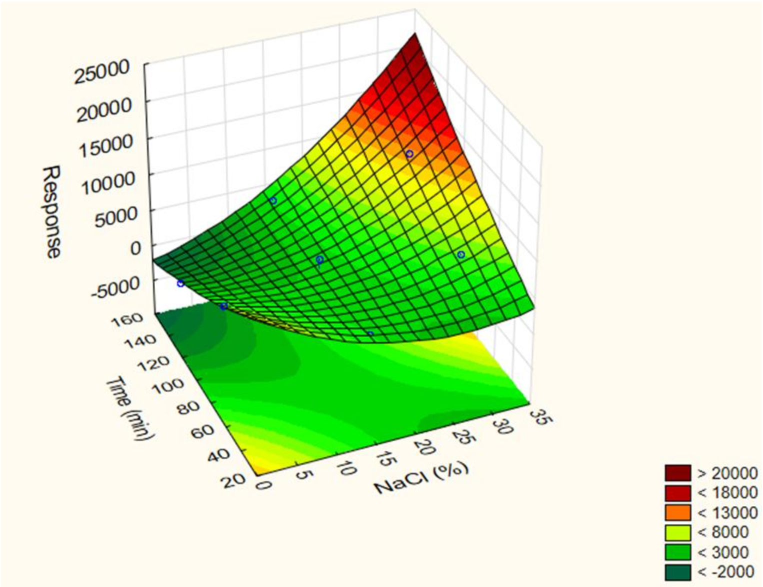

Figure S4. Linearity graphs of Phorate, Alpha-BHC, Aldrin, Endosulfan I (alpha), Dieldrin, 4,4-DDE, and 4,4-DDD.

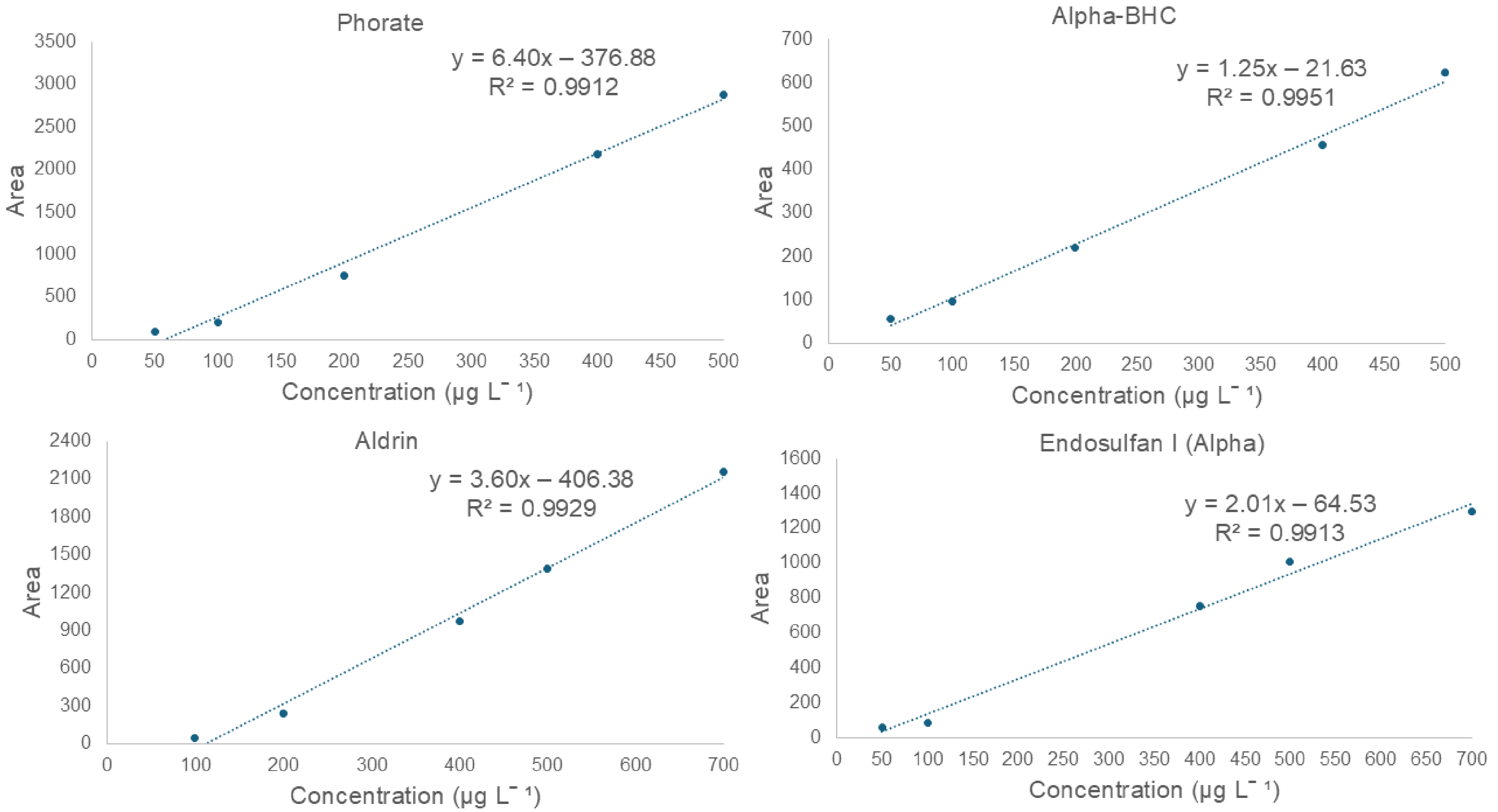

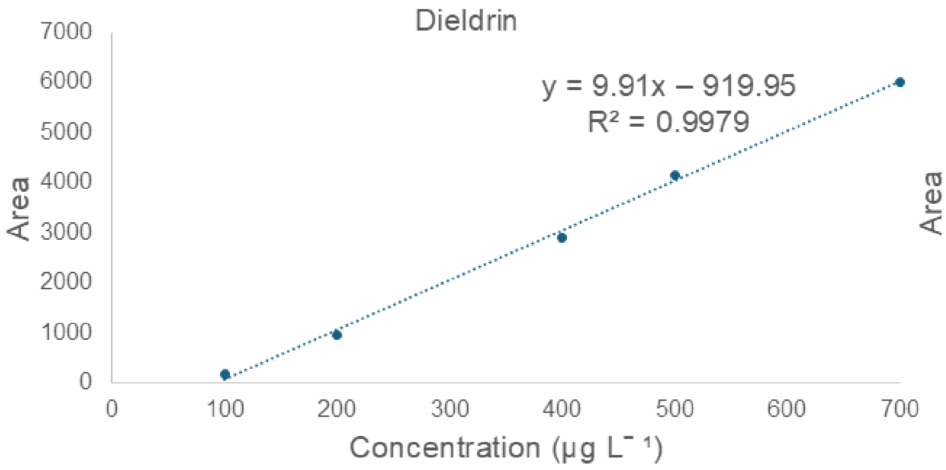
Dieldrin
y = 9.91x – 919.95
R² = 0.9979
Area
7000
6000
5000
4000
3000
2000
1000
0
0
100
200
300
400
500
600
700
Concentration (µg L⁻¹)

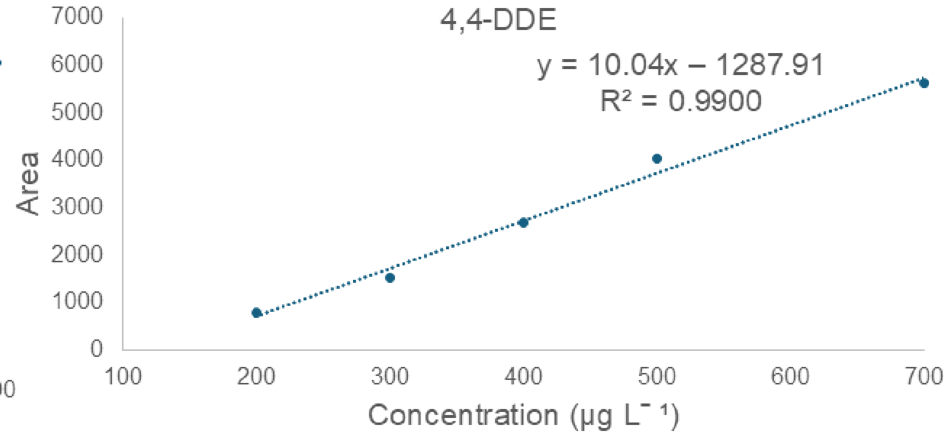
4,4-DDE
y = 10.04x – 1287.91
R² = 0.9900
Area
7000
6000
5000
4000
3000
2000
1000
0
100
200
300
400
500
600
700
Concentration (µg L⁻¹)

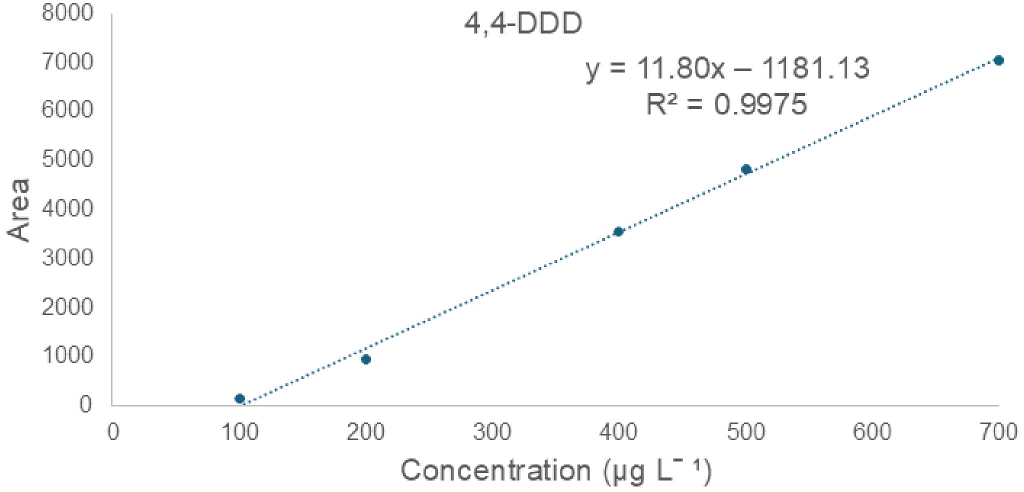
4,4-DDD
y = 11.80x – 1181.13
R² = 0.9975
Area
8000
7000
6000
5000
4000
3000
2000
1000
0
0
100
200
300
400
500
600
700
Concentration (µg L⁻¹)

Figure S5. Storage stability and reusability of TF-CS/MWCNT films evaluated by extraction response after 21 days of storage (A) and after film reuse (B), compared under identical conditions, at a concentration of 200 µg $L^{-1}$.

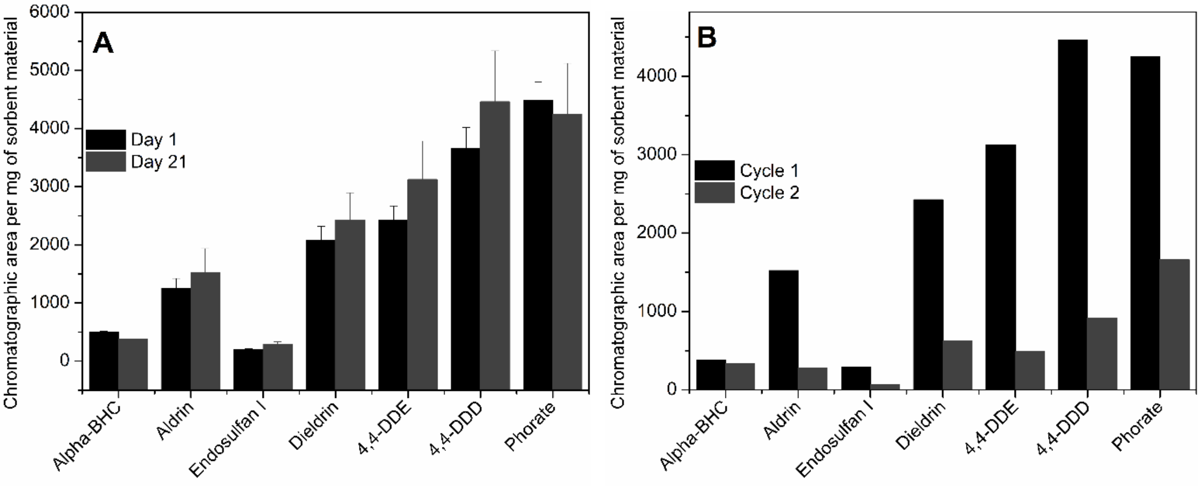

Figure S6. Alpha-BHC, 4,4-DDD, Dieldrin, 4,4-DDE, Endosulfan I (alpha), Aldrin and Phorate chromatograms obtained from real lake water samples (PA and PB). Samples PA-3 and PB-3 were spiked at a concentration of 500 μg $L^{-1}$.

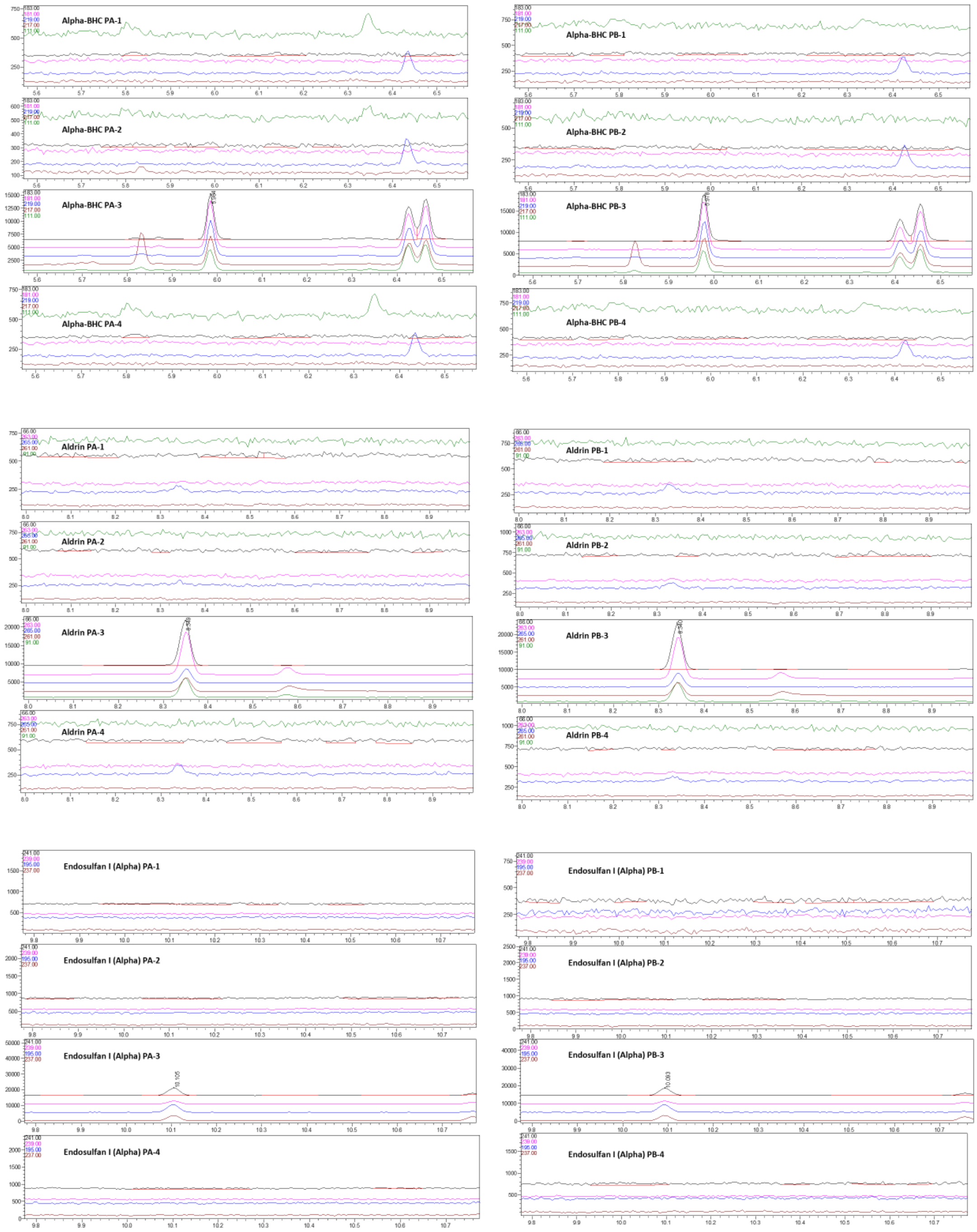

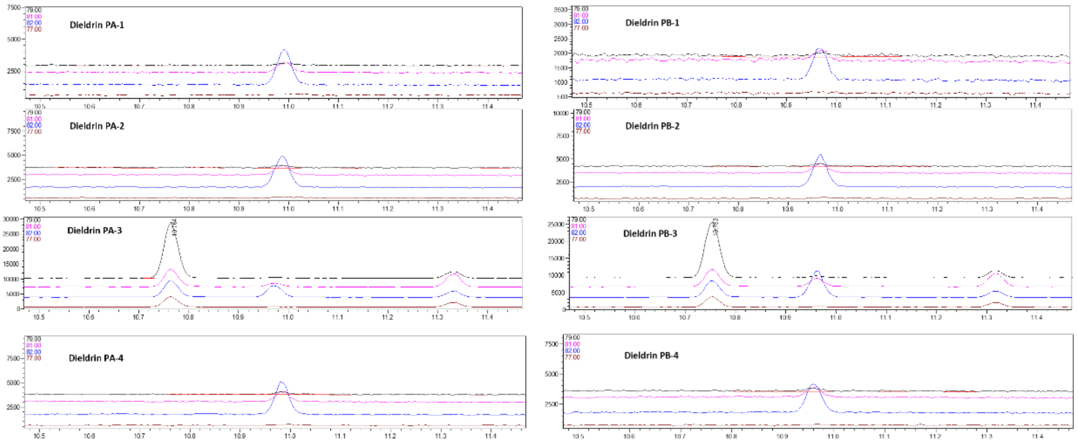
Dieldrin PA-1
Dieldrin PB-1
Dieldrin PA-2
Dieldrin PB-2
Dieldrin PA-3
Dieldrin PB-3
Dieldrin PA-4
Dieldrin PB-4

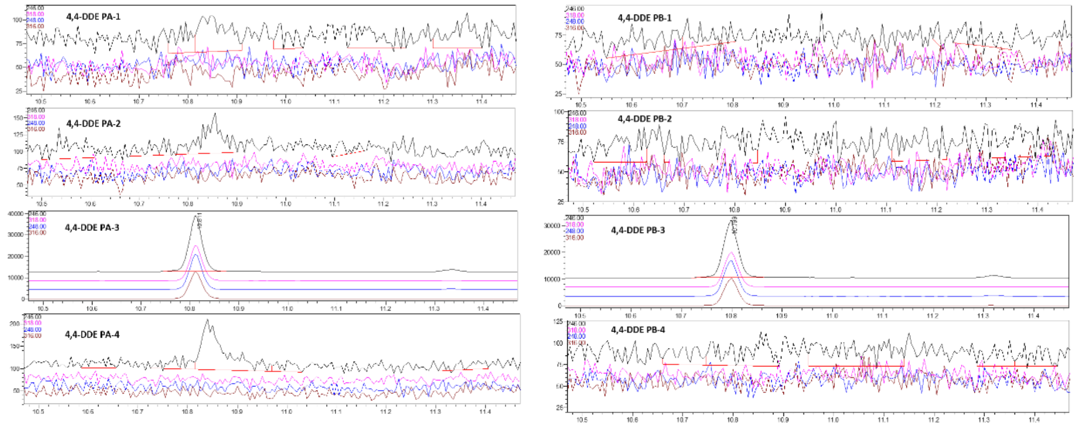
4,4-DDE PA-1
4,4-DDE PB-1
4,4-DDE PA-2
4,4-DDE PB-2
4,4-DDE PA-3
4,4-DDE PB-3
4,4-DDE PA-4
4,4-DDE PB-4

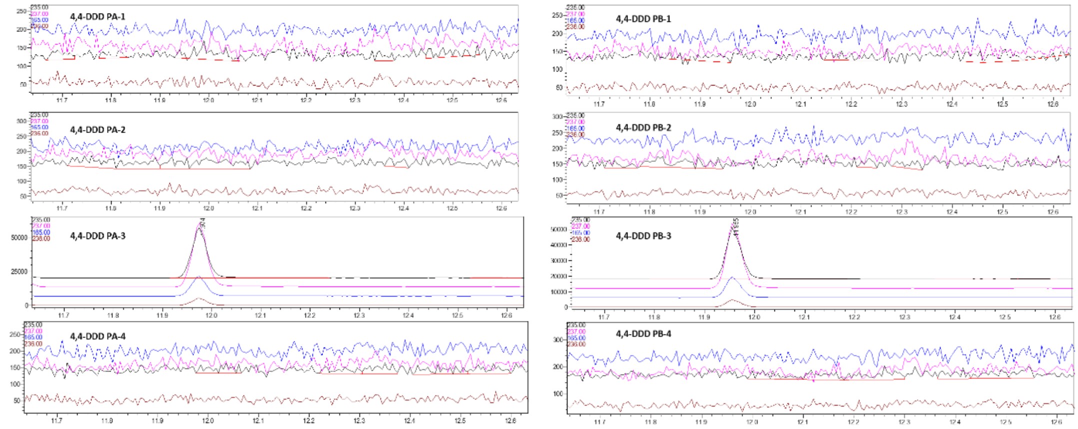
4,4-DDD PA-1
4,4-DDD PB-1
4,4-DDD PA-2
4,4-DDD PB-2
4,4-DDD PA-3
4,4-DDD PB-3
4,4-DDD PA-4
4,4-DDD PB-4

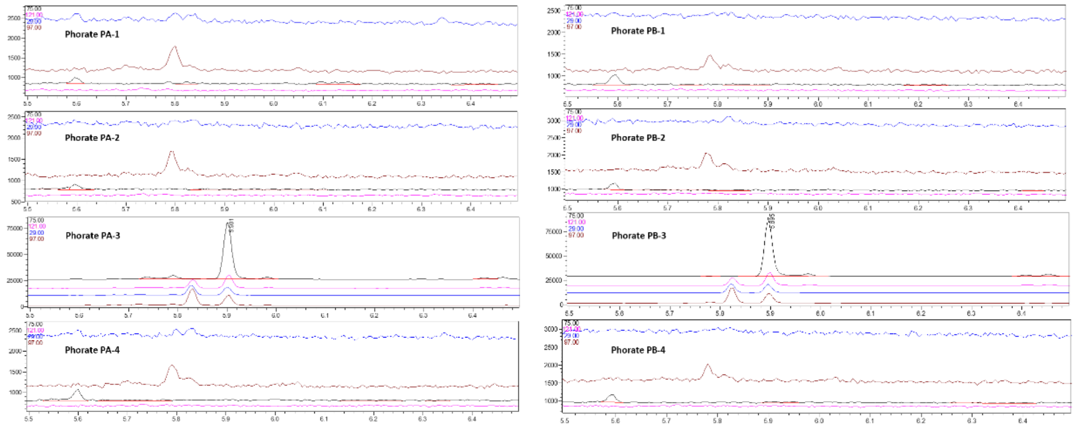
Phorate PA-1
Phorate PB-1
Phorate PA-2
Phorate PB-2
Phorate PA-3
Phorate PB-3
Phorate PA-4
Phorate PB-4
